# Diagnosis and Management of Paget's Disease of Bone in Adults: A Clinical Guideline

**DOI:** 10.1002/jbmr.3657

**Published:** 2019-02-25

**Authors:** Stuart H Ralston, Luis Corral‐Gudino, Cyrus Cooper, Roger M Francis, William D Fraser, Luigi Gennari, Núria Guañabens, M Kassim Javaid, Robert Layfield, Terence W O'Neill, R Graham G Russell, Michael D Stone, Keith Simpson, Diana Wilkinson, Ruth Wills, M Carola Zillikens, Stephen P Tuck

**Affiliations:** ^1^ Centre for Genomic and Experimental Medicine MRC Institute of Genetics and Molecular Medicine University of Edinburgh Edinburgh UK; ^2^ Internal Medicine Department Hospital Universitario Río Hortega University of Valladolid Valladolid Spain; ^3^ MRC Lifecourse Epidemiology Unit University of Southampton Southampton UK; ^4^ Botnar Research Centre, Nuffield Department of Orthopaedics, Rheumatology & Musculoskeletal Sciences University of Oxford Oxford UK; ^5^ Paget's Association Manchester UK; ^6^ Norwich Medical School Faculty of Medicine and Health Sciences University of East Anglia Norwich UK; ^7^ Department of Medicine, Surgery, and Neurosciences University of Siena Siena Italy; ^8^ Hospital Clinic, IDIBAPS, CiberEHD University of Barcelona Barcelona Spain; ^9^ School of Life Sciences University of Nottingham Medical School Queen's Medical Centre Nottingham UK; ^10^ Arthritis Research UK Centre for Epidemiology University of Manchester Manchester UK; ^11^ NIHR Manchester Biomedical Research Centre Manchester University NHS Foundation Trust Manchester Academic Health Sciences Centre Manchester UK; ^12^ The Mellanby Centre for Bone Research University of Sheffield Sheffield UK; ^13^ Bone Research Unit University Hospital Llandough Penarth UK; ^14^ International Medical Press London UK; ^15^ Department of Internal Medicine Erasmus Medical Center Rotterdam the Netherlands; ^16^ Department of Rheumatology The James Cook University Hospital Middlesbrough UK; ^17^ Institute of Cellular Medicine Newcastle University Newcastle upon Tyne UK

**Keywords:** PAGET'S DISEASE OF BONE, ANTIRESORPTIVES, RADIONUCLIDE BONE SCANS, ALP, BISPHOSPHONATES

## Abstract

An evidence‐based clinical guideline for the diagnosis and management of Paget's disease of bone (PDB) was developed using GRADE methodology, by a Guideline Development Group (GDG) led by the Paget's Association (UK). A systematic review of diagnostic tests and pharmacological and nonpharmacological treatment options was conducted that sought to address several key questions of clinical relevance. Twelve recommendations and five conditional recommendations were made, but there was insufficient evidence to address eight of the questions posed. The following recommendations were identified as the most important: 1) Radionuclide bone scans, in addition to targeted radiographs, are recommended as a means of fully and accurately defining the extent of metabolically active disease in patients with PDB. 2) Serum total alkaline phosphatase (ALP) is recommended as a first‐line biochemical screening test in combination with liver function tests in screening for the presence of metabolically active PDB. 3) Bisphosphonates are recommended for the treatment of bone pain associated with PDB. Zoledronic acid is recommended as the bisphosphonate most likely to give a favorable pain response. 4) Treatment aimed at improving symptoms is recommended over a treat‐to‐target strategy aimed at normalizing total ALP in PDB. 5) Total hip or knee replacements are recommended for patients with PDB who develop osteoarthritis in whom medical treatment is inadequate. There is insufficient information to recommend one type of surgical approach over another. The guideline was endorsed by the European Calcified Tissues Society, the International Osteoporosis Foundation, the American Society of Bone and Mineral Research, the Bone Research Society (UK), and the British Geriatric Society. The GDG noted that there had been a lack of research on patient‐focused clinical outcomes in PDB and identified several areas where further research was needed. © 2019 The Authors. *Journal of Bone and Mineral Research* Published by Wiley Periodicals Inc.

## Introduction

Paget's disease of the bone is a nonmalignant skeletal disorder characterized by focal abnormalities in bone remodeling at one (monostotic) or more (polyostotic) skeletal sites. Almost any bone can be affected, but there is a predilection for the pelvis, spine, femur, tibia, and skull.^1^

The main risk factors for PDB include increasing age, male sex, and ethnic background.^2^, ^3^ The risk of developing PDB increases with age, with an approximate doubling in incidence each decade after the age of 50 years.^2^ Paget's is more common in males (1.4:1)^2^ and in certain ethnic groups.^3^ Whites are most commonly affected,^3^ and the disease has been estimated to affect about 1% of people over the age of 55 years in the United Kingdom.^2^ It is also common in other European countries such as France, Spain, and Italy and in people of European descent who have emigrated to other regions of the world, such as Australia, New Zealand, the United States of America, and Canada.^3^ Paget's disease is rare in Scandinavian countries, the Indian subcontinent, and Asian countries. Archeological studies of skeletal remains suggest that these differences in prevalence could be consistent with PDB having arisen as the result of genetic mutations that predispose to the disease in people from North‐West Europe many centuries ago, with spread to other regions of the world through emigration.^4^

At a cellular level, PDB is characterized by increased numbers and activity of osteoclasts coupled with an increase in osteoblast activity.^5^ Bone formation is increased but disorganized, with formation of woven bone, which is mechanically weak and subject to deformity and fracture. The focal increases in osteoclast and osteoblast activity in PDB are also accompanied by marrow fibrosis and increased vascularity of bone. The pathogenesis of PDB is incompletely understood, but genetic factors play a key role. Many affected individuals have a family history,^6^, ^7^ and an autosomal dominant pattern of inheritance with incomplete penetrance may be observed.^8^, ^9^, ^10^ The most important susceptibility gene for PDB is *SQSTM1*,^11^, ^12^ which encodes p62, a protein involved in the nuclear factor kappa B (NF‐κB) signaling pathway.^13^ Mutations in *SQSMT1* have been identified in 40% to 50% of familial cases and in 5% to 10% of patients who do not report having a family history.^9^, ^14^, ^15^ Most of the causal mutations impair the ability of p62 to bind ubiquitin, and this leads to activation of receptor activator of nuclear kappa B ligand (RANKL)‐induced NF‐κB signaling with increased osteoclast activity.^16^ Rarely, familial PDB or PDB‐like disorders may occur in association with mutations in other genes.^17^, ^18^, ^19^ In some of these syndromes, PDB is part of a multisystem disorder accompanied by myopathy and neurodegeneration.^20^, ^21^ Several other common risk alleles have been identified through genomewide association that increase susceptibility to PDB but that in themselves are not causal.^22^

Environmental factors also play a role in PDB as evidenced by the fact that reductions in prevalence and severity have been observed in many countries over the past 25 years, most marked in regions that previously had a high prevalence.^3^, ^23^, ^24^, ^25^, ^26^, ^27^, ^28^, ^29^ In keeping with this, the prevalence of osteosarcoma in adults (a complication of PDB) has also declined in recent years.^30^, ^31^ Various environmental triggers for PDB have been suggested, including dietary calcium or vitamin D deficiency and exposure to environmental toxins,^32^, ^33^ repetitive biomechanical loading or skeletal trauma,^34^, ^35^ and slow virus infections.^36^ The most widely studied environmental factor is slow virus infection, and over the years, measles,^36^ respiratory syncytial virus,^37^ and canine distemper^38^ have all been implicated and overexpression of measles virus nucleocapsids protein in experimental models has been shown to increase bone remodeling.^39^ Attempts to detect evidence of paramyxovirus nucleic acids and proteins in patient material have yielded conflicting results, however,^40^, ^41^, ^42^, ^43^, ^44^, ^45^, ^46^, ^47^ and serological studies have found no evidence of an enhanced immune response to paramyxoviruses in PDB.^48^ It has been reported that the nuclear inclusion bodies that were identified in PDB many decades ago and thought to be measles virus nucleocapsids^36^ are morphologically distinct from measles on ultrastructural analysis.^43^ Experimental evidence has been gained to suggest that they may instead be abnormal protein aggregates due to defects in the autophagy pathway.^49^

Many of the clinical features and complications of PDB are thought to be due to the abnormalities of bone remodeling that are characteristic of the disease. The enlarged bones may cause hearing loss, basilar invagination of the skull, obstructive hydrocephalus, spinal canal stenosis, and paraplegia. The increased vascularity of bone can result in excessive blood loss should orthopedic surgery be required. It has been suggested that in some cases, paraplegia may be due to a vascular “steal” phenomenon, rather than direct compression of the spinal cord by bone enlargement.^50^ High‐output cardiac failure due to increased bone blood flow has been reported but is extremely rare.^51^ The overall frequency with which complications occur in PDB is unknown because it has been estimated that fewer than 10% of patients with X‐ray evidence of PDB come to medical attention.^2^ In those that do present clinically, bone pain is the most common symptom, which was reported to occur in 73% of patients in a recent systematic review.^52^ The mechanisms of pain in PDB are incompletely understood. Although pain in some patients is due to increased metabolic activity, there is a weak correlation between the presence of bone pain and metabolic activity in PDB, at least as reflected by total ALP concentrations. For example, in the study of Reid and colleagues,^53^ 23 of 55 (41.8%) patients with a raised total ALP did not experience bone pain. Similarly, in the PRISM study,^54^ 635 patients had a raised ALP at the baseline visit but 295 (46.4%) of these individuals did not have bone pain. Aside from pain, many other complications of PDB are recognized. In the systematic review cited previously,^52^ bone deformity was present in 21.5% of patients at first presentation, followed by deafness (8.9%) and pathological fracture (8.5%). Osteoarthritis is a common complication of PDB. An analysis of the UK General Practice Research Database in 2002 revealed that patients who have been diagnosed with PDB were more likely to require hip arthroplasty for osteoarthritis compared with age‐matched controls (odds ratio [OR] = 3.1, 95% confidence interval [CI] 2.4–4.1).^2^ Osteosarcoma is a rare complication of PDB, which affects about 0.3% of patients.^2^ It has a poor prognosis even with aggressive treatment.^55^ Giant cell tumor (GCT) is a very rare complication in PDB. A systematic review identified 117 cases of GCT associated with PDB that had been reported in the literature worldwide.^56^ In this series, there was overrepresentation of people of Italian descent from the region of Campania. A high proportion of patients from this region who have GCT and PDB carry a specific missense mutation in the *ZNF678* gene.^57^ In Italy, the prevalence of GCT complicating PDB is estimated to be about 0.8% (L Gennari, unpublished data) but is likely to be much lower in other countries.

### Need for the guideline

The Paget Association and other supporting organizations identified a need for a new guideline that was evidence based, patient focused, and that considered all of the available evidence. This guideline differs from previous guidelines published on this subject^58^, ^59^, ^60^ in that we considered both pharmacological and nonpharmacological treatment options; in that we had patient representation on the guideline development group and sought feedback from patients in the peer‐review process; and in that we have provided information on the key questions used to develop the guideline, as well as details of the search strategy and numbers of publications that were reviewed for each key question.

### Remit of the guideline

The remit of the guideline was to provide patient‐centered, evidence‐based recommendations for the diagnosis and management of classical PDB in adults. The guideline focused on classical PDB and did not consider the diagnosis or management of rare PDB‐like syndromes.

We evaluated tools for the diagnosis of PDB and evaluation of disease extent, the effects of bisphosphonates and other drug treatments on various clinical outcomes, the predictors of treatment response, and the effects of nonpharmacological treatments. Because of limitations in the evidence base, we were unable to evaluate how well imaging techniques and biochemical tests performed in differentiating PDB from other conditions such as hyperostosis frontalis interna, chronic nonbacterial osteomyelitis, and osteosclerotic metastases or in evaluating the clinical role and performance of invasive techniques like bone biopsy in differential diagnosis. That being said, clinical experience indicates that PDB can usually be differentiated quite easily from other conditions by the patient's clinical characteristics and the typical appearances of the disease on radiographic and scintigraphic examination.^61^

The guideline will be of interest to rheumatologists, endocrinologists, physicians involved in care of older people, orthopedic surgeons, internal medicine specialists, metabolic medicine specialists, radiologists, general practitioners, specialist nurses, clinical biochemists, rehabilitation specialists, physiotherapists, occupational therapists, and pharmacists who are involved in the care of patients with PDB. Patients affected by PDB, their caregivers, and other family members may also find the guideline to be of interest.

It should be noted that adherence to the recommendations may not ensure a successful outcome in every case, nor should they be construed as including all proper methods of care or excluding other acceptable methods of care aimed at achieving the same result. The ultimate judgement must be made by the appropriate health care professional(s) responsible for clinical decisions regarding a particular clinical procedure or treatment plan. This judgement should only be arrived at after discussion of the options with the patient, covering the diagnostic and treatment choices available. It is advised, however, that significant departures from guidelines should be fully documented in the patient's medical records at the time the relevant decision is taken.

Recommendations within this guideline are based on the best available clinical evidence. Some recommendations may include the prescription of medicines for which they do not have marketing authorization (a product license). Medicines may be prescribed outside their product license in some countries, and this can be necessary for a variety of reasons such as if the clinical need cannot be met by licensed medicines. In such cases, off‐label prescribing may be employed, provided it is supported by clinical evidence and experience.

## Methods

The Guideline Development Group (GDG) was established in January 2016 by the UK Paget's Association, the European Calcified Tissues Society, and the International Osteoporosis Foundation, which incorporated a multidisciplinary panel of medical practitioners with experience in rheumatology, endocrinology, internal medicine, clinical biochemistry, a nonclinical scientist, a specialist nurse, and one lay member (a patient with PDB). All members were volunteers and none received payment for their participation.

The GDG identified six relevant key questions (KQ) (Supplemental Appendix S1) and used the 2013 update of GRADE methodology (https://gdt.gradepro.org/app/handbook/handbook.html) to assess the strength of evidence and to formulate recommendations.^62^, ^63^, ^64^, ^65^

A literature search based on each of the KQ was performed according to GRADE recommendations. Search strategies and flow diagrams for each search are provided in Supplemental Appendix S2. The initial search was performed in August 2016 supervised by Dr Ruth Wills from the medical communications company International Medical Press (http://www.intmedpress.com). We incorporated search findings from the 2017 Cochrane review,^66^ which focused on bisphosphonate treatment of PDB in March 2017. The search was updated in January 2018 but no new articles of relevance to the KQ were identified. We initially searched for systematic reviews that addressed the KQ followed by randomized controlled trials if no systematic reviews were available. If no randomized controlled trials had been performed, we searched for observational studies and case series, provided the number of individuals studied was greater than 10. Individual case reports and case series of fewer than 10 subjects were generally excluded, unless these provided insights into the questions that were not addressed by larger studies or clinical trials. The summary of findings in these articles were used to grade the quality of evidence. For other interventions and diagnostic tests, the panel conducted their own review by assessing the articles that were relevant to the question and excluding articles that were not. Significant limitations were found when dealing with diagnostic tests for PDB because most studies were performed in patients known to have PDB. Because of this, there were very few reliable studies that could be used to establish the accuracy of different diagnostic tests. The GDG noted that PDB does not have a single gold standard test for diagnosis, since both X‐rays and radionuclide bone scans can provide different information that often can be considered diagnostic of PDB.

The members of the GDG assessed the quality of the evidence according to the methodology described by the GRADE system. In this system, quality of supporting evidence is assessed based on explicit methodological criteria and classified as “high” (further research is very unlikely to change our confidence in the estimate of effect), “moderate” (further research is likely to have an important impact on our confidence in the estimate of effect and may change the estimate), “low” (further research is very likely to have an important impact on our confidence in the estimate of effect and is likely to change the estimate), or “very low” (any estimate of effect is very uncertain).

The method we used for wording of recommendations is shown in Table 1. The GDG considered the quality of evidence, the balance between benefit and harms, patients’ values and preferences, and the resources and potential costs involved. For instances where interventions or investigations were recommended (or not recommended), the GDG felt that the benefits clearly outweighed the harms for most people or vice versa. For instances where there was a closer balance between benefits and harm for interventions and investigations, the GDG made a conditional recommendation. For conditional recommendations, the GDG felt that clinicians should discuss with patients and families the relative merits of alternative management options to the intervention to help each patient arrive at a decision consistent with his or her values and preferences.

**Table 1:** Wording of Recommendations

Recommendation	Language	Meaning for patients	Meaning for clinicians
Positive recommendation	The intervention or investigation is *recommended*.	Most patients would want the intervention or investigation.	Most patients should receive the intervention or investigation.
Negative recommendation	The intervention or investigation is *not recommended*.	Most patients would not want the intervention or investigation.	Most patients should not receive the intervention or investigation.
Conditional recommendation	The intervention or investigation *may be considered*.	Some patients would want the recommended intervention or investigation but others would not.	Different choices may be applicable to different patients depending on their values and preferences. The clinician should discuss the risks and benefits with the patient before reaching a decision.
Insufficient evidence	The intervention or investigation is *not recommended*.	Most patients would not want the intervention or investigation.	Most patients should not receive the intervention or investigation.

For instances where there was insufficient evidence to support the use of an intervention or investigation for a specific indication, it was agreed that a statement should be made to acknowledge that the intervention or investigation was not recommended.

The guideline process was validated in accordance with the Appraisal of Guidelines for Research and Evaluation, using the AGREE reporting checklist 2016.^67^

The draft guidelines were sent to several stakeholders and were externally reviewed by the representatives from the American Society of Bone and Mineral Research, the European Calcified Tissues Society, the International Osteoporosis Foundation, the British Geriatrics Society, the Bone Research Society (UK), as well as several patients who are members of the UK Paget's Association. Several other organizations were invited to comment but did not respond (Supplemental Appendix S3). The final version of the guideline was revised and updated to take account of the comments that were received. The GDG intends to conduct regular reviews every 3 years after publication of the guidance to determine whether the evidence base has progressed significantly enough to alter the current guideline recommendations and require an update.

## Results and Recommendations

In this section, the results of the literature search are summarized, along with the recommendations of the guideline group for each key question that was posed.

### Diagnosis of Paget's disease of bone

The following section deals with techniques used for the diagnosis of PDB. A limitation of the studies described in this section is that with one exception^68^ the literature search failed to identify any studies in which diagnostic tools were assessed or compared in a population‐based setting. Similarly, no studies were identified that addressed the order in which diagnostic tests should be used. In view of this, the present section reports upon the performance of different modalities in evaluating the presence and extent of the disease in patients suspected to have PDB.

### Radiographs

**Figure 1:**
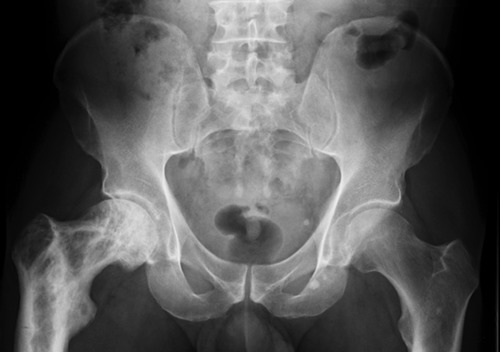
X‐ray features of PDB. Pelvic radiograph from a patient with PDB affecting the upper right femur showing alternating areas of osteolysis and osteosclerosis in the greater and lesser trochanters and femoral neck; loss of distinction between the cortex and medulla in the upper femur; bone expansion and deformity of the affected femur; and a pseudofracture on the lateral aspect of the femur opposite the lesser trochanter.

The radiological features of PDB have been reviewed elsewhere.^69^ The disease has characteristic features on X‐ray that are summarized in Table 2 and illustrated in Fig. 1. Individually, these features are not specific, but when they occur in combination, they are usually diagnostic.

**Table 2:** X‐ray Features of PDB

Osteolytic areas
Cortical thickening
Loss of distinction between cortex and medulla
Trabecular thickening
Osteosclerosis
Bone expansion
Bone deformity

Guañabens and colleagues^70^ investigated the issue of how many regions of the skeleton would need to be X‐rayed to pick up PDB, based on analysis of plain X‐rays and radionuclide bone scans in 208 patients already known to have PDB from a disease registry in Spain. The study showed that compared with bone scan, an abdominal X‐ray (defined as an X‐ray that includes the lower ribs and femoral heads) would pick up PDB in 79% of cases; that addition of an X‐ray or the skull and facial bones would increase the pickup rate to 89%; and that addition of an X‐ray of the upper tibias increased the pickup rate to 93%. The evidence summary and recommendations for the use of X‐rays in the diagnosis and assessment of patients with PDB are shown in Table 3.

**Table 3:** Role of X‐rays in the Diagnosis of PDB

*Risk‐benefit balance*
Plain X‐rays targeted to the abdomen, skull, and facial bones and both tibias are likely to detect 93% of PDB bone lesions compared with 79% for an abdominal X‐ray. The benefit to the patient in making a diagnosis from having additional radiographs is likely to outweigh the risk to the patient in terms of the additional radiation exposure.
*Quality of evidence*
Very low
*Patient values and preferences*
It's likely that the majority of patients would be content with having radiographs of three sites as opposed to one to more accurately make a diagnosis of PDB.
*Costs and use of resources*
Plain X‐rays are widely available and relatively inexpensive.
*Recommendation*
Plain X‐rays of the abdomen, tibias, skull, and facial bones are recommended as an initial diagnostic screening test in patients suspected to have PDB on biochemical or clinical grounds.

### Radionuclide bone scintigraphy

Radionuclide bone scintigraphy is widely considered to be a valuable technique for the diagnosis of PDB and assessment of disease extent.^71^ Radionuclide bone scanning is performed after intravenous injection of the gamma‐emitting isotope Technetium‐99m (Tc^99m^) linked to a bisphosphonate (most commonly as Tc^99m^‐methylene diphosphonate). When PDB involves a bone, the radiolabeled bisphosphonate accumulates in sites where there is high bone remodeling. In PDB, sites of involvement are visualized as a region of intense and homogeneous tracer uptake, which in long bones starts at the metaphysis and extends down the shaft. Although many other conditions such as fibrous dysplasia, infections, metastases, and arthritis can be associated with increased tracer uptake on bone scans, the appearances in PDB usually allow differentiation from other conditions. In certain sites, the scintigraphic features of PDB are highly specific. These are “clover” or “mickey mouse sign” and the “heart sign” when PDB affects the spine.^72^, ^73^ This observation can be of value in the differential diagnosis with vertebral metastases but false‐positive results have been described.^74^ Several investigators have compared the performance of bone scanning with plain X‐rays in the evaluation of PDB. Wellman and colleagues compared the performance of radionuclide bone scans with X‐rays in 108 PDB patients.^75^ They reported that 101 lesions were detected both by X‐rays and radionuclide scans; that a further 36 lesions were detected only on radionuclide scan and not by X‐ray; and that 11 lesions were detected by X‐ray only.^75^ They concluded that radionuclide bone scan was more sensitive than X‐ray in detecting sites of involvement but that scan may be negative in sclerotic (“burned out’) lesions. Another study by Meunier and colleagues^76^ compared the performance of X‐rays and radionuclide scans in 170 PDB patients. They reported evidence of increased tracer uptake in 863 sites of which 16 (1.9%) showed changes consistent with osteoarthritis; 6 (0.7%) with changes consistent with bone metastases, and 3 (0.35%) with changes consistent with vertebral fractures. Of the 838 sites showing scintigraphic changes consistent with PDB, 727 (86.7%) showed evidence of PDB on X‐ray. Seventy‐one (8.4%) showed changes typical of PDB on bone scan only, and in another 23 sites, radiographs were positive and bone scans were negative. The authors reported that of 863 sites detected on bone scan, 30.6% were symptomatic. The authors concluded that bone scans are more sensitive than radiographs at detecting PDB lesions but that bone scans may be negative if the disease is inactive. A further comparative study of 23 patients with PDB showed 127 sites of involvement of which 120 (94.5%) were recognized on scan compared with 94 (74%) on radiological skeletal survey.^77^ Of these, 7 lesions (5.5%) were detected on X‐ray only and 33 (25.9%) on bone scan only; lesions were detected by both modalities in 87 (67.5%) sites. When data from these three studies are combined, 83.5% of bone lesions in PDB were detected by both X‐rays and radionuclide bone scans; 12.8% by bone scan only, and 3.7% by X‐ray only. The evidence summary and recommendations for the use of radionuclide bone scans in the diagnosis and assessment of patients with PDB are shown in Table 4.

**Table 4:** Role of Radionuclide Bone Scans in the Diagnosis of PDB

*Risk‐benefit balance*
Radionuclide bone scans are more sensitive than radiographs at detecting bone lesions in PDB, but radiographic evidence of PDB may be observed in about 3.7% of sites when the bone scan is negative. However, the majority of sites detected by imaging are asymptomatic.
*Quality of evidence*
Very low
*Patient values and preferences*
It is likely that many patients may not object to having a bone scan in addition to targeted radiographs to fully assess the extent of PDB.
*Costs and use of resources*
Radionuclide bone scans are widely available but are more expensive than plain X‐rays.
*Recommendation*
Radionuclide bone scans, in addition to targeted radiographs, are recommended as a means of fully and accurately defining the extent of the metabolically active disease in patients with PDB.

### Magnetic resonance imaging and computed tomography

There have been few studies on the role of magnetic resonance imaging (MRI) and computed tomography (CT) in the diagnosis of PDB. Roberts and colleagues compared the appearances of MRI, CT, and plain radiographs in 13 patients with PDB.^78^ The MRI findings and CT findings were consistent with the abnormalities found in plain radiographs. Another study compared CT appearances of the skull in 10 patients with PDB with those in 10 patients with fibrous dysplasia (FD) of the skull.^79^ The authors identified 10 differentiating features including a ground glass appearance (favoring FD), symmetric cranial involvement (PDB), thick cortices (PDB) and involvement of the sinuses, sphenoid, orbit, and nasal cavity (all favoring FD).

Although the use of MR imaging and CT imaging is not generally indicated for the diagnosis of PDB, clinical experience indicates that MRI and/or CT imaging is very useful for the investigation of several complications of PDB, including basilar invagination, spinal stenosis, and osteosarcoma.^78^ The evidence summary and recommendations for the use of MRI scans and CT scans in the diagnosis and assessment of patients with PDB are shown in Table 5.

**Table 5:** Role of MRI and CT Scanning in the Diagnosis of PDB

*Risk‐benefit balance*
The radiation exposure with CT scans is higher than plain X‐rays or radionuclide bone scans, but MRI scans do not involve radiation exposure.
*Quality of evidence*
Very low
*Patient values and preferences*
Patients with claustrophobia may prefer to avoid MRI.
*Costs and use of resources*
Both CT scans and MRI scans are considerably more expensive than plain X‐rays or radionuclide bone scans.
*Recommendation*
There was insufficient evidence to recommend MRI or CT imaging for the diagnosis of PDB and neither technique is recommended for this purpose. These imaging techniques are recommended for the assessment of disease complications.

### Biochemical markers

The elevations in bone remodeling that are characteristic of active PBD can be detected clinically by measurement of biochemical markers of bone turnover in blood and urine samples. It should be noted, however, that elevations in markers of bone turnover occur in many disease states and cannot be used in isolation for the diagnosis of PDB.

The most widely used biochemical marker for the diagnosis of PDB is serum total alkaline phosphatase (ALP), which is usually performed as part of liver function tests in a routine biochemistry screen. A population‐based study by Eekhof^(68)^ specifically looked at the performance of ALP in detecting PDB in participants of the Rotterdam study in Holland. The researchers selected 105 individuals from a cohort of 4406 subjects who had an elevated total ALP with normal transaminases and matched these subjects with 625 controls who had a normal total ALP. They found that the relative risk for PDB (based on radiographs of the hands, spine, pelvis, and knees) in the presence of a raised total ALP was 10.9 (95% CI 4.8–24.9) in men and women older than 55 years of age.^68^ This is the only study that has addressed the accuracy of any biochemical marker for the diagnosis of PDB. It showed that the sensitivity of total ALP was 57.7% (95% CI 38.9–74.5); specificity 88.9% (95% CI 85.9–91.3); positive likelihood ratio 5.19 (95% CI 3.45–7.82); and negative likelihood ratio 0.48 (95% CI 0.30–0.75). It should be noted that the calculated sensitivity may be an underestimate because radiographic assessment of PDB in the study didn't include the skull and some patients with PDB of this site could have been missed. It's also important to emphasize that of 26 patients with radiological features of PDB, only 11 (42%) had elevated total ALP concentrations.

The performance of various biochemical markers of bone turnover in patients with PDB was studied by Alvarez in 51 patients who had not received treatment in the previous 6 months. This showed that of the markers studied, procollagen type I N‐terminal propeptide (PINP) showed the highest proportion of increased values among bone formation markers when compared with BALP and total ALP (94%, 82%, and 76%, respectively).^80^ In the same study, urinary cross‐linked N‐terminal telopeptide of type I collagen (uNTX) values were increased in 96% of patients with PDB compared with urinary pyridinoline (uPYD) (69%), urinary deoxypyridinoline (uDPD) (71%), and urinary cross‐linked beta C‐terminal telopeptide of type I collagen (uβCTX) (65%). Osteocalcin (OC) was increased in only 34% of patients. Another study by the same group of researchers^81^ evaluated total ALP and bone alkaline phosphatase (BALP) in a series of 59 patients with PDB who had been untreated for at least 6 months, along with various other markers including the carboxy‐terminal propeptide of type I collagen (PICP), tartrate‐resistant acid phosphatase (TRAP), telopeptide carboxyterminal propeptide of type I collagen (ICTP), urinary pyridinoline (PYD), and deoxypyridinoline D‐PYR) and hydroxyproline (HYP). Total ALP values were elevated in 74.5% of patients, but BALP was elevated in 90%. The best‐performing resorption marker was D‐PYD, which was elevated in 73%. Of the 15 subjects with normal total ALP, serum BALP was increased in 60%.

Woitge and colleagues reported that uNTX values were increased in 94% of a small series of 18 PDB patients compared with 64% for serum cross‐linked beta C‐terminal telopeptide of type I collagen (sβCTX).^82^ In the same study, total ALP was increased in 100% of cases.^82^

The role of biochemical markers in predicting disease extent was evaluated in a systematic review by Al‐Nofal and colleagues.^83^ This study synthesized data from 17 observational studies and 1 randomized trial in patients with PDB. The markers included were serum total ALP, BSALP, uNTX, uβCTX, sβCTX, and PINP. In treatment‐naïve patients, circulating concentrations of all markers were found to be highly significantly associated with extent of PDB as determined by radionuclide bone scintigraphy. The correlation between marker concentrations and disease extent for individual markers were BSALP = 0.750 (95% CI 0.621–0.839); PINP = 0.756 (95% CI 0.692–0.809); total ALP = 0.617 (95% CI 0.518–0.700), sβCTX = 0.583 (95% CI 0.324–0.761); uβCTX = 0.589 (95% CI 0.332–0.765); and uNTX = 0.796 (95% CI 0.702–0.862). The *p* value for differences between the individual markers was not significant (*p* = 0.083).

The evidence summary and recommendations for the use of biochemical markers in the diagnosis and assessment of patients with PDB are shown in Table 6.

**Table 6:** Role of Biochemical Markers in the Diagnosis of PDB

*Risk‐benefit balance*
The risk of having to provide blood or urine samples for diagnosis is minimal and outweighed by the benefit of making a correct diagnosis.
*Quality of evidence*
Very low
*Patient values and preferences*
Most patients are unlikely to be concerned about providing a blood or urine sample.
*Costs and use of resources*
Serum total ALP is widely available and considerably cheaper than other biochemical markers that have been assessed in PDB.
*Recommendation*
Serum total ALP is recommended as a first‐line biochemical screening test in combination with liver function tests in screening for the presence of PDB. If total ALP values are normal and clinical suspicion of metabolically active PDB is high, measurement of BALP, PINP, or uNTX may be considered to screen for metabolically active disease.

### Effects of drug treatment in Paget's disease

This section focuses on drug treatment for PDB and the effects of treatment on complications and clinical features of the disease. Two broad categories of drug treatment are commonly used in patients with PDB. Specific anti‐Pagetic treatment involves the use osteoclast inhibitors to reduce the elevations in bone turnover that are characteristic of active disease, whereas other treatments such as analgesics, nonsteroidal anti‐inflammatory drugs, and anti‐neuropathic agents are used for symptom control.^54^, ^84^ We have mainly focused on the use of bisphosphonates, which are currently considered to be the treatment of choice for PDB and which are the only agents that have been evaluated in randomized controlled trials.^66^

Through the literature review, we identified studies of several osteoclast inhibitors that had been employed at some point in the treatment of PDB, including glucagon,^85^ mithramycin,^86^ actinomycin D,^87^ and gallium nitrate.^88^ These were not considered further because the guideline group felt they were of historical interest and were evaluated in uncontrolled studies with no comparator. Similarly, supportive treatments such as nonsteroidal anti‐inflammatory drugs, anti‐neuropathic agents, and analgesics are widely used for pain control in patients with PDB, and in the PRISM study, at least one of these agents was used by all patients.^54^ We did not identify any trials in which the effectiveness of these agents was investigated specifically in PDB. The effects of denosumab^89^ and calcitonin^90^ in PDB were also reviewed and are discussed separately within this article.

### Bone pain

Bone pain in PDB is a complex symptom that is associated with increased bone turnover but that may also occur in patients without increased metabolic activity. This section focuses on the effects of bisphosphonates in the treatment of bone pain. Although other drugs such as analgesics, nonsteroidal anti‐inflammatory drugs, and anti‐neuropathic agents are often used in the management of bone pain associated with PDB, these agents haven't been investigated in controlled clinical trials. Calcitonin and denosumab have also been reported to improve bone pain in PDB but are discussed separately because neither has been investigated in randomized trials.

A meta‐analysis of placebo‐controlled studies with various bisphosphonates^66^ involving a total of 418 subjects showed that these drugs were effective at reducing bone pain compared with placebo such that the proportion of patients who achieved any reduction in bone pain was 45% versus 23% (relative risk [RR] = 1.97, 95% CI 1.29–3.01; number needed to treat [NNT] = 5, 95% CI 2–15). It should be noted that all but one of these trials^53^ involved etidronate^91^, ^92^, ^93^ or tiludronate,^94^, ^95^, ^96^ which are no longer in widespread use.

A randomized open‐label trial involving 89 subjects^97^ showed that a single intravenous infusion of 4 mg zoledronic acid was more likely to give pain relief than 30 mg intravenous pamidronate when given on 2 consecutive days every 3 months (RR = 1.30, 95% CI 1.10–1.53; NNT = 5, 95% CI 3–11). A randomized open‐label trial comparing intravenous pamidronate 60 mg intravenously every 3 months with oral alendronic acid 40 mg daily in 3‐month blocks reported no difference in bone pain between the treatments, although the article did not include detailed information on this outcome.^98^ Another randomized double‐blind study involving 357 patients published by Reid and colleagues^99^ showed that zoledronic acid given as a single dose of 5 mg intravenously was more likely to give pain relief than risedronate sodium 30 mg daily orally for 2 months (RR = 1.36, 95% CI 1.06–1.74; NNT = 7, 95% CI 4–24).

An insight into the durability of the response of pain with different bisphosphonates comes from an extension of the Reid study,^100^ which compared the effects of a single dose of zoledronic acid 5 mg with a single 2‐month course of risedronate sodium 30 mg daily. The extension study focused on a subgroup of 267 individuals in whom total ALP values were normal at the end of the core study. Clinical relapse, as defined by recurrence of bone pain, occurred in 14 of 152 (9.2%) patients in the zoledronic acid group compared with 29 of 115 (25.2%) in the risedronate sodium group. It should be noted that the rate of clinical relapse was more than 10 times greater than the rate of biochemical relapse in the zoledronic acid group (0.7%) and was about 25% greater than the rate of biochemical relapse in the risedronate sodium group (20%). This indicates that biochemical relapse of PDB and clinical relapse as defined by the recurrence of pain are distinct entities.

The evidence summary and recommendations for the use of bisphosphonates to treat bone pain in PDB are shown in Table 7.

**Table 7:** Effect of Bisphosphonate Treatment on Bone Pain

*Risk‐benefit balance*
Bisphosphonates improve bone pain in PDB compared with placebo and comparative studies within bisphosphonates have shown that zoledronic acid is more likely to give an improvement than pamidronate and risedronate sodium. These bisphosphonates have a generally favourable adverse effect profile. In addition, most patients required other pain‐relieving medications such as analgesics and nonsteroidal anti‐inflammatory drugs for pain control.
*Quality of evidence*
Moderate
*Patient values and preferences*
Most patients that have bone pain are likely to favor the potential benefits of bisphosphonates with or without other analgesics considering their generally favorable adverse event profile.
*Costs and use of resources*
Bisphosphonates are inexpensive, but intravenous therapy involves additional support costs and costs in terms of patient time attending for the infusion that need to be considered.
*Recommendation*
Bisphosphonates are recommended for the treatment of bone pain associated with Paget's disease. Zoledronic acid is recommended as the bisphosphonate most likely to give a favorable pain response.

### Health‐related quality of life

No information was available from randomized trials with which to evaluate the effects of bisphosphonates on health‐related quality of life compared with placebo.

A comparison of zoledronic acid with risedronate sodium^99^ showed that the average physical component summary score of SF‐36 improved to a greater extent in the zoledronic acid group, although the absolute difference was about 2 points, which is below the 5‐point threshold that is considered clinically significant.^99^ When the SF36 physical summary score data were analyzed by multivariate testing taking baseline scores into account, the authors reported a nominally significant difference (*p* = 0.04) favoring zoledronic acid, but this was not adjusted for multiple comparisons. When the data were expressed as the proportion of patients whose SF36 physical summary score improved after treatment, the difference favored zoledronic acid (RR = 1.30, 95% CI 1.18–1.42).^66^ In an extension of the same study,^100^ the mean change from baseline SF36 summary score favored zoledronic acid (mean difference 3.8, 95% CI 3.12–4.49). However, this analysis was not intention to treat, and was based on a selected group of patients who had normal total ALP values at the end of the core study.

The evidence summary and recommendations for the use of bisphosphonates to improve quality of life in PDB are shown in Table 8.

**Table 8:** Effect of Bisphosphonate Treatment on Health‐Related Quality of Life

*Risk‐benefit balance*
We found no evidence to evaluate the effects of bisphosphonates on quality of life compared with placebo. We found evidence that zoledronic acid improved some aspects of quality of life more than risedronate sodium, but the differences were below the threshold that is considered clinically significant.
*Quality of evidence*
Very low
*Patient values and preferences*
Quality of life is important to patients. If treatment strategies could be identified that offered a significant improvement in quality of life, it is likely that they would be favored by patients.
*Costs and use of resources*
Bisphosphonates are inexpensive, but intravenous therapy involves additional support costs and costs in terms of patient time attending for the infusion that need to be considered.
*Recommendation*
There is insufficient evidence that bisphosphonate therapy improves quality of life to a clinically meaningful extent in PDB, and they are not recommended for this indication.

### Prevention of fractures

Fractures in patients with PDB can be divided into two categories: those that occur in affected bone (pathological fractures) and those that occur in unaffected bone. The vast majority of pathological fractures in PDB affect the femur or tibia. The literature review revealed that there was insufficient evidence to evaluate the effects of bisphosphonates on incident fractures of either category when compared with placebo. A Cochrane review^66^ of placebo‐controlled trials reported that information on fracture was only available in 356 participants, and the reports did not distinguish pathological fractures from fractures in unaffected bone. In these studies, the rate of fractures in the placebo group was 0 of 79 (0%) versus 4 of 277 (1.4%) in the bisphosphonate group (RR = 0.89, 95% CI 0.18–4.31). There was no data on which to evaluate the effects of different individual bisphosphonates on incident fractures or to evaluate the effects of bisphosphonates on fractures in bone affected by PDB.

The evidence summary and recommendations for the use of bisphosphonates to prevent fractures in PDB are shown in Table 9.

**Table 9:** Effect of Bisphosphonate Treatment on Fracture Prevention

*Risk‐benefit balance*
The effects of bisphosphonates on prevention of fractures in PDB have not been adequately studied.
*Quality of evidence*
Very low
*Patient values and preferences*
Prevention of fractures is valued by patients with PDB. If treatment strategies could be identified that were effective in preventing fractures, it is likely that they would be favored by patients.
*Costs and use of resources*
Bisphosphonates are inexpensive, but intravenous therapy involves additional support costs and costs in terms of patient time attending for the infusion that need to be considered.
*Recommendation*
There is insufficient evidence that bisphosphonate therapy prevents fractures in PDB, and they are not recommended for this indication.

### Progression of osteoarthritis

There was no evidence upon which to evaluate the effects of bisphosphonates on progression of osteoarthritis compared with placebo, and no evidence to evaluate the effects of individual bisphosphonates compared with one another on progression of osteoarthritis.

The evidence summary and recommendations for the use of bisphosphonates to prevent progression of osteoarthritis in PDB are shown in Table 10.

**Table 10:** Effect of Bisphosphonate Treatment on Progression of Osteoarthritis

*Risk‐benefit balance*
The effects of bisphosphonates on progression of osteoarthritis have not been adequately studied.
*Quality of evidence*
Very low
*Patient values and preferences*
Prevention of osteoarthritis is likely to be valued by patients with PDB. If treatment strategies could be identified that were effective in preventing progression of osteoarthritis, it is likely that they would be favored by patients.
*Costs and use of resources*
Bisphosphonates are inexpensive, but intravenous therapy involves additional support costs and costs in terms of patient time attending for the infusion that may need to be considered.
*Recommendation*
There is insufficient evidence that bisphosphonate therapy prevents progression of osteoarthritis in PDB, and they are not recommended for this indication.

### Progression of hearing loss

The effects of treatment on hearing loss is considered separately from neurological symptoms for two reasons: the first is that it has been studied separately and the second is that in many cases deafness is not due to nerve compression but is a conductive deafness possibly related to abnormalities in the temporal bone.^101^ There was no evidence from randomized trials upon which to evaluate the effects of bisphosphonates compared with placebo; no evidence to compare the effects of individual bisphosphonates; and no evidence to compare the effects of other treatments with bisphosphonates or other treatments with placebo. We identified one observational study of 25 PDB patients^102^ in which the effects of tiludronate (400 mg daily for 3 months; *n* = 15) or pamidronate (30 mg i.v. for 6 days; *n* = 10) on hearing loss were studied in patients with PDB of the skull. Audiometry demonstrated sensorineural hearing loss in 12 patients, conductive hearing loss in 4, and a mixed pattern in 6 patients. The authors reported no significant change in hearing thresholds after 12 months overall, although they commented that there was a nonsignificant (7.5 db) increase in hearing thresholds in the high‐frequency region in those with sensorineural loss.^(102)^

The evidence summary and recommendations for the use of bisphosphonates to prevent progression of hearing loss in PDB are shown in Table 11.

**Table 11:** Effect of Bisphosphonate Treatment on Progression of Hearing Loss

*Risk‐benefit balance*
The effects of bisphosphonates on progression of hearing loss have not been adequately studied.
*Quality of evidence*
Very low
*Patient values and preferences*
Prevention of progression of hearing loss is likely to be valued by patients with PDB. If treatment strategies could be identified that were effective in preventing progression of hearing loss, it is likely that they would be favored by patients.
*Costs and use of resources*
Bisphosphonates are inexpensive, but intravenous therapy involves additional support costs and costs in terms of patient time attending for the infusion that may need to be considered.
*Recommendation*
There is insufficient evidence that bisphosphonate therapy prevents progression of hearing loss in PDB, and they are not recommended for this indication.

### Blood loss during elective orthopedic surgery

There was no evidence from randomized trials upon which to evaluate the effects of bisphosphonates compared with placebo, the effects of individual bisphosphonates, or the effects of other treatments on operative blood loss during elective surgery. Some information was available from observational studies on the relation between having received anti‐Pagetic treatment and operative blood loss. Wegrzyn reviewed the outcome of 39 cementless hip replacements in a series of 32 patients undergoing surgery in a French center between 1992 and 2006.^103^ All patients received intravenous pamidronate before surgery and 31 of 39 (79%) hip replacements had been performed in patients with a normal total ALP at the time of surgery. The average blood loss was 744 mL (range 250–2000 mL), which the authors commented was greater than in patients undergoing similar procedures that did not have PDB (range 200–450 mL). Gabel^104^ studied blood loss in 13 patients who had 16 total knee replacements (TKR) at a single US center between 1974 and 1986. The average blood loss was 481 mL (range 100–2000) and the authors commented that there was no difference between blood loss in patients who had previous treatment with either calcitonin or etidronate or those who did not have treatment. Similar findings were reported by Lee in 21 TKR from 20 patients referred to a US center between 1978 and 1999.^105^ Blood loss was estimated as 300 mL (range 100–600 mL), but the authors found no difference between blood loss in patients who had or had not received preoperative treatment with etidronate (278 mL versus 315 mL, *p* = 0.32).^105^ A systematic review conducted by Jorge‐Mora and colleagues reviewed the effects of anti‐Pagetic therapy on blood loss and other outcomes after elective spinal surgery in 17 case reports.^106^ The most common indications for surgery were spinal cord compression (*n* = 8), spinal stenosis (*n* = 6), and back pain (*n* = 3). Bisphosphonate was given before the surgery in 7 patients, but the type of bisphosphonate used and the dose were not recorded. Bleeding was noted as a complication in 0 of 7 patients given bisphosphonate and 4 of 10 patients not given bisphosphonate (*p* = 0.22, Fisher's exact test). Parvizi and colleagues reported upon the influence of treatment on blood loss during osteotomy in 22 PDB patients.^107^ Calcitonin was given to 6 patients and pamidronate to 3 patients before surgery. The authors commented that excessive bleeding was observed in all cases but did not define what was meant by excessive bleeding. They also commented that medical treatment significantly reduced intraoperative blood loss and that estimated blood loss was higher in patients with active disease but no data on blood loss or disease activity in these subgroups were provided.

The evidence summary and recommendations for the use of bisphosphonates to prevent or reduce blood loss during elective orthopedic surgery are shown in Table 12.

**Table 12:** Effect of Bisphosphonate Treatment on Blood Loss During Elective Orthopedic Surgery

*Risk‐benefit balance*
The data on blood loss in patients who have and have not had bisphosphonate treatment before elective orthopedic or spinal surgery are conflicting and difficult to interpret.
*Quality of evidence*
Very low
*Patient values and preferences*
Prevention of blood loss during surgery is likely to be valued by patients with PDB. If treatment strategies could be identified that were effective in preventing blood loss during elective orthopedic surgery, it is likely that they would be favored by patients.
*Costs and use of resources*
Bisphosphonates are inexpensive, but intravenous therapy involves additional support costs and costs in terms of patient time attending for the infusion that may need to be considered.
*Recommendation*
There is insufficient evidence that bisphosphonate therapy reduces perioperative blood loss during elective orthopedic surgery, and they are not recommended for this indication.

### Bone deformity

There was no evidence from randomized trials upon which to evaluate the effects of bisphosphonates compared with placebo, the effects of individual bisphosphonates, or the effects of other treatments on the prevention or treatment of bone deformity. One case series of 9 PDB patients with facial deformity was identified.^108^ Each of these patients was treated with etidronate or clodronate for between 1 and 6 years, and facial deformity was measured using a stereophotogrammetric technique. Based on this analysis, the authors reported that facial deformity (as reflected by a derived measure of facial or skull volume) improved in 7 of 8 cases.

The evidence summary and recommendations for the use of bisphosphonates to prevent progression of bone deformity in PDB are shown in Table 13.

**Table 13:** Effect of Bisphosphonate Treatment on Bone Deformity

*Risk‐benefit balance*
The effects of bisphosphonates on bone deformity have not been adequately studied.
*Quality of evidence*
Very low
*Patient values and preferences*
Bone deformity is of concern to patients. If treatment strategies could be identified that were effective in preventing bone deformity, it is likely that they would be favored by patients.
*Costs and use of resources*
Bisphosphonates are inexpensive, but intravenous therapy involves additional support costs and costs in terms of patient time attending for the infusion that may need to be considered.
*Recommendation*
There is insufficient evidence that bisphosphonates can prevent or treat bone deformity in PDB, and they are not recommended for this indication.

### Neurological symptoms

This section concerns the effect of treatment on neurological symptoms other than deafness, which was considered earlier. No randomized comparative trials were identified in which the effects of bisphosphonates or other treatments have been evaluated in respect to neurological symptoms. We identified two case series of patients that addressed this issue. Chen and colleagues described the response to treatment with salmon or porcine calcitonin given subcutaneously in 49 PDB patients with neurological symptoms treated between 1969 and 1973 at a single referral center in the US.^109^ The starting dose was 100 IU by subcutaneous injection, although subsequently the dose was reduced in some patients to 50 IU 3 times weekly. Treatment was continued for 7 to 31 months (average 23 months). The indication for treatment in 10 patients was cranial nerve lesions (other than lesions of the 8th cranial nerve), spinal nerve root dysfunction in 15, spinal cord problems in 6, and miscellaneous neurological problems in 8. The authors reported objective improvement in 40% of patients with a cranial nerve lesion responded to treatment, as compared with 33% with spinal nerve problems, 50% with spinal cord symptoms, and 0% with miscellaneous problems. In another case series from the UK, Douglas reported the results of treatment with calcitonin, etidronate, or clodronate in 8 patients with neurological dysfunction due to Paget's disease of the spine.^50^ Seven of the 8 patients were treated with calcitonin 100 IU daily and all improved neurologically. One patient treated with clodronate also improved. In 3 patients whose symptoms recurred despite treatment with calcitonin, there was a response to etidronate or clodronate. Douglas also reviewed the results of medical treatment of spinal dysfunction from other published case reports with calcitonin at that time^50^ and identified 13 additional patients who had been treated with calcitonin for spinal cord dysfunction whose symptoms had improved after therapy.

The evidence summary and recommendations for the use of calcitonin and bisphosphonates in the treatment of neurological symptoms in PDB are shown in Table 14.

**Table 14:** Effect of Calcitonin and Bisphosphonates on Neurological Symptoms

*Risk‐benefit balance*
Most experience in the medical treatment of spinal cord dysfunction in PDB comes from case series of patients treated with calcitonin, and clinical benefit from treatment has been reported in a proportion of treated patients. Similar benefit has been noted in a small number of patients treated with bisphosphonates.
*Quality of evidence*
Very low
*Patient values and preferences*
Spinal cord dysfunction and the symptoms associated with this complication is of major concern to patients. Treatment strategies that are effective in preventing spinal cord dysfunction are likely to be favored by patients.
*Costs and use of resources*
Calcitonin is a relatively expensive treatment that needs to be administered by injection. Bisphosphonates are inexpensive but have been little studied in this situation. Intravenous bisphosphonate therapy involves additional support costs and costs in terms of patient time attending for the infusion that may need to be considered.
*Recommendation*
A trial of calcitonin treatment may be considered as part of the treatment package in patients with PDB who have evidence of neurological dysfunction. Bisphosphonate treatment may also be considered, although there are few studies to support the use of bisphosphonates in this situation.

### Treatment of increased metabolic activity in asymptomatic patients

Bisphosphonates are highly efficacious at reducing the elevations in bone turnover that are characteristic of active PDB. Here we focus on the effects of treatment on serum total ALP because it is the most commonly used biochemical marker of metabolic activity in PDB and has served as the primary outcome measure in clinical trials where bisphosphonates have been compared with placebo and with other bisphosphonates. In a Cochrane review,^66^ it was noted that bisphosphonates achieved a 50.1% (95% CI 32.5–67.7) greater reduction in total ALP (592 participants) than placebo and the RR of bisphosphonates normalizing total ALP was 9.96 (95% CI 3.74–26.58). In the same review, a comparison of nitrogen‐containing bisphosphonates with non‐nitrogen‐containing bisphosphonates showed that nitrogen‐containing bisphosphonates were more effective at normalizing total ALP than non‐nitrogen‐containing bisphosphonates (212 participants) (RR = 4.3, 95% CI 2.72–6.79; NNT = 2, 95% CI 1–4). Within the nitrogen‐containing bisphosphonates, zoledronic acid was more efficacious at reducing total ALP than pamidronate (90 participants) or risedronate sodium (347 participants) (RR = 2.57, 95% CI 1.79–3.70; NNT = 2, 95% CI 1–3 and RR = 1.53, 95% CI 1.33–1.76; NNT = 3; 95% CI 3–5, respectively.

The duration of effect of different bisphosphonates on serum total ALP concentrations has also been studied. A randomized trial comparing oral risedronate sodium 30 mg daily for 2 months with oral etidronate 400 mg daily for 6 months showed that total ALP values remained suppressed in 53% of the risedronate sodium group compared with 14% of the etidronate group.^110^ In a long‐term extension of the HORIZON Paget's study,^(100)^ 88% of patients treated with a single dose of 5 mg zoledronic acid intravenously still had a normal serum total ALP after 5 years’ follow‐up compared with 47% of patients treated with oral risedronate sodium. It should be noted that the attrition rate in this study was high and that only patients with normal ALP at the end of the core study were eligible to be enrolled into the extension.

Although many clinical trials of bisphosphonates have enrolled patients on the basis that serum total ALP values are elevated (whether or not symptoms were present), we found no clinical trials or observational studies that specifically addressed the issue of whether treatment of asymptomatic patients with bisphosphonates that have metabolically active PDB was of benefit in preventing complications of the disease.

Of some relevance to the issue of treating asymptomatic patients is the fact that bisphosphonates can promote healing of lytic lesions at least in the short term. In one observational study of PDB patients treated with pamidronate, healing of lytic lesions was demonstrated in some cases at 6 months, but longer‐term follow‐up of these patients after 2 years showed progression of lytic lesions once again, even though biochemical markers of bone turnover were normal at this point.^111^ The effects of bisphosphonates on lytic lesions have been studied in two randomized controlled trials. One examined the effects of alendronic acid 40 mg daily for 6 months in 55 PDB patients compared with placebo. The average age was about 70 years; 19% had previously been treated with anti‐Pagetic medication and 32 (58%) had bone pain thought to be due to PDB at baseline. The authors reported healing of lytic lesions in 11 of 23 (47.8%) patients treated with alendronic acid and no change in 12 of 23 (52.1%) patients. Corresponding values in placebo‐treated patients were 1 in 23 healed and no change in 22 of 23 patients (95.6%). Bone biopsies were obtained through Paget's bone in 4 alendronic acid–treated patients and 9 placebo‐treated patents. Histomorphometry showed lower bone turnover in the alendronic acid–treated cases. Another randomized trial compared the effects of alendronate 40 mg daily for 6 months with etidronate 400 mg daily for 6 months in 89 PDB patients of average age about 70 years.^112^ It showed that lesions improved in 32.4% of the ALN group, whereas 8.8% showed worsening. The corresponding proportions in the etidronate group were 26.5% and 14.7%, respectively, a difference that was not significant.

The issue of giving bisphosphonates with the aim of suppressing bone turnover in established PDB was addressed by the PRISM trial, which is discussed in more detail later. The GDG noted that risks and benefits of giving prophylactic zoledronic acid to asymptomatic people at risk of developing PDB was being addressed by the ZiPP study (EUDRACT 2008‐005667‐34), which is due to report in 2020.

The evidence summary and recommendations for the use of bisphosphonates with the primary aim of supressing bone turnover symptoms in asymptomatic patients with PDB are shown in Table 15.

**Table 15:** Effects of Bisphosphonate Treatment on Asymptomatic Patients With Increased Metabolic Activity

*Risk‐benefit balance*
Bisphosphonates are highly effective at reducing metabolic activity in PDB as reflected by concentrations of total ALP and other biochemical markers of bone turnover. Improvements in lytic lesions have also been reported in short‐term studies. The clinical benefit of giving bisphosphonates in asymptomatic patients with the primary aim of supressing metabolic activity is unknown.
*Quality of evidence*
High
*Patient values and preferences*
Patients with PDB who have elevated concentrations of total ALP or other biochemical markers of bone turnover in the absence of symptoms may or may not derive clinical benefit from treatment. Some patients may favor treatment, whereas others may not in view of the potential risk of adverse effects and uncertain benefit.
*Costs and use of resources*
Bisphosphonates are inexpensive, but intravenous therapy involves additional support costs and costs in terms of patient time attending for the infusion that need to be considered.
*Recommendation*
Bisphosphonate therapy may be considered to suppress metabolic activity in PDB, but the clinical benefit is uncertain. Within this class of drugs, nitrogen‐containing bisphosphonates are more effective than non‐nitrogen‐containing bisphosphonates, and within the bisphosphonates, zoledronic acid is most efficacious.

### Neoplastic transformation

There was no evidence from randomized trials upon which to evaluate the effects of bisphosphonates compared with placebo; the effects of individual bisphosphonates; or the effects of other treatments on the prevention of osteosarcoma or GCT. Similarly, no observational studies were identified that evaluated the effects of treatment on neoplastic transformation.

The evidence summary and recommendations for the use of bisphosphonates with aim of preventing neoplastic transformation in PDB are shown in Table 16.

**Table 16:** Effect of Bisphosphonate Treatment on Neoplastic Transformation

*Risk‐benefit balance*
The effects of bisphosphonates on the prevention of neoplastic transformation in PDB have not been adequately studied.
*Quality of evidence*
Very low
*Patient values and preferences*
Prevention of neoplastic transformation is likely to be highly valued by patients with PDB. Treatment strategies that are effective in preventing neoplastic transformation would most likely be favored by patients.
*Costs and use of resources*
Bisphosphonates are inexpensive, but intravenous therapy involves additional support costs that need to be considered.
*Recommendation*
There is insufficient evidence to show that bisphosphonates prevent neoplastic transformation in PDB, and they are not recommended for this indication.

### Adverse events

This section evaluates the adverse events that have been reported with bisphosphonate treatment with an emphasis of those of relevance to the treatment of PDB. Atypical femoral fractures, uveitis, osteonecrosis of the jaw, hypocalcemia, and impaired renal function are recognized to be rare adverse effects of bisphosphonates. A recent Cochrane review^66^ evaluated the frequency of rare adverse events in PDB patients treated with bisphosphonates by reviewing the websites of the Food and Drug Administration (FDA), the Medicines and Healthcare products Regulatory Agency (MHRA), the European Medicines Agency (EMA), and the Australian Regulatory Agency (AARB). The estimated frequency of osteonecrosis of the jaw (ONJ) in people with PDB receiving oral bisphosphonates was estimated as between 0.0004% and 0.06%, which is much lower than in osteoporosis. There is no clear evidence regarding the risk of ONJ after use of intravenous bisphosphonates for PDB, although one case was reported in the PRISM‐EZ study in a patient who received intensive bisphosphonate therapy.^113^ Atypical femoral fractures (AFF) are thought to be a class effect of bisphosphonates; as of 2017, the EMA had received only one report of an AFF in a patient with PDB. The authors of the Cochrane review speculated that the infrequent occurrence of ONJ and AFF in PDB might be related to the fact that patients tend to have intermittent or short‐term courses for treatment of the disease.

In a recent Cochrane review,^66^ no statistically significant difference was found in adverse effects with oral bisphosphonates compared with placebo (6 studies, 678 participants, risk difference 0.11, 95% CI 0.00–0.22). Similarly, the risk of discontinuation due to adverse events was similar compared with placebo (517 participants; RR = 1.01, 95% CI 0.38–2.69). It should be noted that these comparisons predominantly involved non‐nitrogen‐containing oral bisphosphonates. Zoledronic acid was found to have an increased risk of adverse effects when compared with placebo (RR = 2.57, 95% CI 1.21–5.44). The most common adverse event in studies with zoledronic acid was a transient flu‐like illness.^99^ The prevalence and severity of this adverse effect has not been studied in detail in PDB, but in osteoporosis, it was estimated to occur in 42.5% of patients; of these episodes, 46% were considered to be mild by the investigator, 45% moderate, and 10% severe.^114^ There is good evidence that the flu‐like symptoms are milder after second and subsequent infusions of zoledronic acid compared with the first infusion.^114^

The evidence summary and recommendations with regard to adverse effects of bisphosphonate treatment are shown in Table 17.

**Table 17:** Adverse Events of Bisphosphonate Treatment

*Risk‐benefit balance*
Serious adverse events with bisphosphonates are rare. In PDB, oral bisphosphonates have a similar adverse event profile as placebo but that a transient flu‐like illness occurs commonly with zoledronic acid. Usually this is of mild to moderate severity but can be severe in some patients.
*Quality of evidence*
Very low
*Patient values and preferences*
Adverse events are of concern to patients and a proportion of individuals may decline treatment because of the risk of adverse events.
*Costs and use of resources*
Bisphosphonates are inexpensive, but intravenous therapy involves additional support costs that may need to be considered.
*Recommendation*
We recommend that patients undergoing treatment with bisphosphonates for PDB are informed about their favorable adverse event profile. We also recommend that patients are advised that a transient flu‐like illness occurs commonly with intravenous zoledronic acid.

### Treatment strategy in Paget's disease

This section focuses on randomized trials that have compared different treatment strategies in PDB. Only three studies were identified that directly addressed this issue. These were the PRISM study^54^ and its extension^113^ and the study of intravenous versus intramuscular neridronate.^115^ All three studies concerned the use of bisphosphonates.

### Treatment of increased metabolic activity or symptoms?

The Paget's Disease, Randomized Trial of Intensive versus Symptomatic Management (PRISM) study compared the effects of a treat to target strategy aimed at normalizing total ALP compared with a strategy aimed at controlling symptoms^54^ in 1324 patients with PDB. The average age of participants at entry to the study was about 74 years with an average disease duration of 8 years. About 70% of patients had previously been treated with bisphosphonates; about 47% had elevated total ALP values at baseline, and 46% had bone pain thought to be caused by PDB. There was a poor correlation between presence of bone pain thought to be due to PDB and an elevated ALP value, however.^54^ Participants randomized to receive “intensive” bisphosphonate treatment (*n* = 661) were prescribed bisphosphonates with the aim of maintaining or suppressing total ALP values to within the reference range irrespective of whether bone pain was present. Risedronate sodium was the bisphosphonate of first choice, but any licensed bisphosphonate could be used. In the symptomatic group (*n* = 663), the therapeutic goal was to control bone pain. This was initially attempted using analgesics, but if the response was inadequate, non‐nitrogen‐containing bisphosphonates or calcitonin were used first followed by nitrogen‐containing bisphosphonates if necessary. The primary endpoint was clinical fracture. Secondary endpoints included fractures through Pagetic bone, orthopedic procedures, quality of life assessed by SF36, HAQ and EQ5D, bone pain, bone deformity, progression of hearing loss in patients with skull involvement assessed by audiometry, and adverse events. PRISM was an event‐driven study, which was stopped when 95 clinical fractures occurred. The average duration or follow‐up was 3 years with a range of 2 to 4 years.

The PRISM‐extension with zoledronic acid study (PRISM‐EZ) employed the same strategy as in PRISM, but zoledronic acid was used as the treatment of first choice in the intensive arm.^113^ Patients within PRISM‐EZ maintained the same treatment allocation as they had been randomized to in PRISM. The PRISM‐EZ study followed 270 patients in the intensive group and 232 in the symptomatic group, providing an average total duration of 7.3 years follow‐up since the beginning of the PRISM study. The primary and secondary endpoints were the same in PRISM‐EZ as in PRISM, except that patients did not undergo audiometry in the extension.

#### Fractures

The number of clinical fractures in the intensive and symptomatic PRISM treatment arms were similar. In the intensive group, 46 of 661 (7.0%) participants had clinical fractures compared with 49 of 663 (7.3%) in the symptomatic group (RR = 0.94, 95% CI 0.64–1.39). Fractures through Pagetic bone occurred in 8 of 661 (1.2%) of the intensive group and 13 of 663 (2.0%) of the symptomatic group (RR = 0.62, 95% CI 0.22–1.60). In the PRISM‐EZ trial,^113^ 22 of 270 (8.1%) of participants in the intensive group had clinical fractures compared with 12 of 232 (5.2%) in the symptomatic group (RR = 1.84, 95% CI 0.76–4.44). Fractures through Pagetic bone occurred in 5 of 270 (1.9%) in the intensive group versus 2 of 232 (0.9%) in the symptomatic group (RR = 2.15, 95% CI 0.42–10.96).

#### Orthopedic surgery

In the PRISM study, the number of patients undergoing orthopedic surgery in the intensive treatment group was 48 of 661 (7.2%) and 55 of 663 (8.2%) in the symptomatic group (RR = 0.88, 95% CI 0.60–1.27). Of the 103 procedures performed, 73.7% were joint replacements for osteoarthritis. In the PRISM‐EZ study, 15 of 270 (5.5%) of patients in the intensive group underwent orthopedic surgery compared with 7 of 232 (3.0%) patients in the symptomatic group (RR = 1.84, 95% CI 0.76–4.44). Joint replacements were also the most common orthopedic procedure in PRISM‐EZ and were more commonly required in the intensive group 11 of 270 (4.1%) versus 4 of 232 (1.7%).

#### Health‐related quality of life

The PRISM study showed no significant difference in health‐related quality of life between the treatment groups at any time point using various tools including SF36, EQ5D, and HAQ. Within the PRISM‐EZ study, small differences in some aspects of quality of life were observed between the treatment groups at some time points, but the differences were below the 5‐point threshold that is considered clinically significant and were not consistently observed at different time points.

#### Bone pain

The PRISM study showed no difference between treatment groups in the proportion of patients with bone pain at 2 years (311 of 422 [73.7%] versus 295 of 423 [69.7%], *p* = 0.20) or bone pain thought by the clinician to be due to PDB (96 of 311 [30.8%] versus 78 of 295 [26.4%], *p* = 0.22). In the PRISM‐EZ study, there were no differences in bone pain or bone pain thought by the clinician to be due to PDB except at 2 years where the standardized mean difference, calculated by propensity scoring, showed 1.3% fewer patients with bone pain (95% CI 0.3–2.3) in the intensive treatment group.

#### Progression of deafness

The PRISM study showed no significant difference between treatment groups in progression of hearing loss, as determined by audiometry and the proportion of patients using a hearing aid over an average of 3 years of follow‐up. Audiometry showed that the mean (±SD) change in hearing threshold was +1.8 ± 14.6 in the left ear in the intensive group compared with 0 ± 12.6 in the symptomatic group (mean, 95% CI difference = 1.8, –3.4 to 7.0). Corresponding values in the right ear were 2.5 ± 5.7 versus 2.1 ± 9.4 (mean, 95% CI difference = 0.5, –2.5 to 3.3). At the baseline visit of PRISM, 151 of 663 (22.9%) of the symptomatic group and 144 of 661 (21.9%) of the intensive group used a hearing aid. The proportion of hearing aid users increased to a similar extent in both groups such that by the end of study 133 of 486 (27.3%) of the symptomatic group used a hearing aid compared with 134 of 505 (26.5%) of the intensive group.

#### Adverse events

In the PRISM study, the numbers of adverse events and serious adverse events in the two treatment groups were similar. In the PRISM‐EZ study, the number of patients with adverse events in the intensive group was 226 of 270 (83.7%) compared with 196 of 232 (84.5%) in the symptomatic group (RR = 0.99, 95% CI 0.92–1.08). The number of serious adverse events in the two treatment groups was 87 of 270 (32.2%) versus 66 of 232 (28.4%) (RR = 1.28, 95% CI 0.96–1.72).

#### Alkaline phosphatase

In the PRISM study, serum concentrations of total ALP were significantly lower in the intensive group from 4 months onward. At the end of the study, 78.8% of the intensive group had a total ALP within the reference range compared with 61.2% of the symptomatic group (*p* < 0.001). In the PRISM‐EZ study, total ALP values were lower at baseline and throughout the study in the intensive group. By the end of the study, total ALP values were within the reference range in 85.3% of the intensive group versus 70.3% of the symptomatic group (*p* < 0.001).

The evidence summary and recommendations with regard to employing a strategy of supressing bone turnover as the primary therapeutic goal in PDB as opposed to treating symptoms are shown in Table 18.

**Table 18:** Treating Symptoms or Increased Metabolic Activity with Bisphosphonates in PDB

*Risk‐benefit balance*
A strategy of intensive bisphosphonate therapy aimed at maintaining total ALP concentrations within the reference range performed similarly to a strategy of treatment with bisphosphonates and other drugs that aimed to control symptoms, with respect to the occurrence of clinical fractures, fractures through Pagetic bone, requirement for orthopedic surgery, quality of life, bone pain, and progression of hearing loss.
*Quality of evidence*
Moderate
*Patient values and preferences*
Prevention of fractures and orthopedic procedures, and improvements in bone pain, quality of life, and prevention of progressive hearing loss are all highly valued by patients.
*Costs and use of resources*
Bisphosphonates are inexpensive drugs, but intravenous therapy may involve additional support costs that may need to be considered. More frequent courses of therapy increase health care costs and resources as compared with less frequent courses of treatment.
*Recommendation*
Treatment aimed at improving symptoms is recommended over a treat‐to‐target strategy aimed at normalizing total ALP in PDB.

### Route of administration of bisphosphonates

The literature review identified several studies in which different modes of administration of bisphosphonates for the treatment of PDB were investigated, but the only randomized trial was by Merlotti and colleagues with neridronate, a nitrogen‐containing bisphosphonate licensed in Italy for PDB.^115^ The study group was composed of 57 patients with active PDB as defined by a serum total ALP value above the upper limit of the reference range. All patients were reported to have bone pain before treatment. Participants were randomized to receive intravenous neridronate (100 mg i.v. on 2 consecutive days) or intramuscular neridronate (25 mg once weekly for 8 weeks). The primary endpoint was normalization of total ALP. Secondary endpoints included bone pain and the time taken until ALP normalized. Normalization of ALP levels at 6 months was achieved in 24 of 27 patients (88.9%) in the intravenous group and 26 of 29 patients (89.6%) in the intramuscular group. Longer‐term follow‐up at 36 months revealed that normal total ALP values were maintained in 13 of 27 (48.1%) and 13 of 29 (44.8%) of patients in the intravenous and intramuscular groups, respectively. Pain had improved or disappeared in 21 of 27 (77%) of patients given intravenous therapy at 6 months compared with 19 of 29 (65.5%) given intramuscular therapy, a difference that was not significant (chi‐square 1.02, *p* = 0.30). Adverse effects in the two treatment groups were similar. The authors concluded that both routes of administration gave equivalent therapeutic responses but commented that the intramuscular route was slightly more expensive (115 versus 90 euros).

The evidence summary and recommendations with regard to administering neridronate intravenously as opposed to intramuscularly are shown in Table 19.

**Table 19:** Route of Administration of the Bisphosphonate Neridronate in PDB

*Risk‐benefit balance*
Information from randomized trials is only available for the comparison of intravenous and intramuscular modes of administration of neridronate. Both routes of administration were found to give similar results in terms of suppression of ALP and control of bone pain.
*Quality of evidence*
Low
*Patient values and preferences*
Improvements in bone pain are valued by patients. Some patients might prefer two infusions as opposed to eight intramuscular injections, although the intramuscular route could be preferred in patients with poor venous access.
*Costs and use of resources*
Neridronate is inexpensive with little difference between regimens. Nursing support costs may be higher with intramuscular therapy, but day patient facilities and other support costs may be higher with intravenous therapy.
*Recommendation*
For patients with metabolically active PDB with bone pain treated with neridronate, either the intravenous or intramuscular route can be recommended.

### Calcitonin

Calcitonin was one of the first osteoclast inhibitors to be used in the treatment of PDB. No randomized trials were identified in which the effects of calcitonin were compared with placebo or with other osteoclast inhibitors. One of the largest case series of patients treated with calcitonin was published by Martin and colleagues, who reported on the response to porcine calcitonin 80 MRC units daily in a case series of 38 patients with active PDB who received 44 courses of treatment by daily injection for periods of between 6 weeks and 18 months.^116^ Bone pain improved in 32 of 38 (81.8%) patients after treatment, although the method of assessing bone pain was not described. Serum total ALP concentrations also decreased from a mean (SEM) of 899 (145) U/L to 579 (130) U/L (*p* < 0.001). The reference range for total ALP wasn't provided and so it was impossible to determine in what proportion of patients total ALP values had fallen to within the reference range. Six patients (15.7%) were reported to have adverse effects, the most common of which were nausea and diarrhea. In one patient (2.6%), treatment was stopped because of adverse effects and in one (2.6%) the dose was reduced because of adverse effects. Since these early reports, long‐term calcitonin therapy for osteoporosis has been associated with an increased risk of certain cancers. We identified one randomized trial of 44 patients with active PDB that had bone pain inadequately unresponsive to analgesia who were randomized to receive oral etidronate in a dose of 400 mg daily for 6 months or oral etidronate 400 mg daily plus calcitonin, 100 IU three times weekly by subcutaneous injection.^117^ The response of biochemical markers of bone turnover in these patients was compared with a group of historical controls with PDB who had been treated with calcitonin alone at the same dose. In the historical controls treated with calcitonin, total ALP decreased from an average of 1261 U/L before treatment to 595 U/L 6 months after treatment (53% reduction, *p* < 0.001). Corresponding values for the etidronate group were 1228 U/L to 539 U/L (56% reduction, *p* < 0.001) and for the etidronate plus calcitonin group 1448 U/L to 428 U/L (71% reduction, *p* < 0.001). The combination of etidronate plus calcitonin was more effective at decreasing total ALP than etidronate alone (*p* < 0.002). The authors did not specifically comment on the effects of these agents on bone pain in the results section. Calcitonin has been studied in case series of patients with neurological dysfunction associated with PDB and treatment has been associated with clinical benefit in some cases (Table 14).

The evidence summary and recommendations with regard to the use of calcitonin in the treatment of metabolic activity and pain in PDB are shown in Table 20.

**Table 20:** Effects of Calcitonin on Bone Pain and Metabolic Activity in PDB

*Risk‐benefit balance*
Calcitonin improves bone pain in PDB and decreases total ALP concentrations. Long‐term administration of calcitonin has been associated with an increased risk of cancer.
*Quality of evidence*
Very low
*Patient values and preferences*
Improvements in bone pain are highly valued by patients. Adverse events may be observed with calcitonin, and the need for repeated injections at frequent intervals may be considered a barrier by some patients.
*Costs and use of resources*
Calcitonin is considerably more expensive than bisphosphonates.
*Recommendation*
Calcitonin may be considered for the short‐term treatment of bone pain in PDB where bisphosphonates are contraindicated.

### Denosumab

There have been two case reports in the use of denosumab 60 mg by subcutaneous injection every 6 months in PDB in patients where bisphosphonates were poorly tolerated or contraindicated. In both cases, denosumab resulted in a decrease in total ALP concentrations and an improvement of bone pain.^89^, ^118^ Three open‐label trials have been conducted to study the effects of denosumab in the treatment of GCT, but PDB was an exclusion in two of these studies^119^, ^120^ and in the third, no information on co‐existing PDB was available.^121^ The posology in this situation is an initial loading dose of 120 mg denosumab subcutaneously two times weekly followed by 120 mg 4 times weekly thereafter. Of three case reports where denosumab was given to PDB patients with non‐resectable GCT, the treatment improved bone pain and reduced tumor size.^122^, ^123^, ^124^

The evidence summary and recommendations with regard to the use of denosumab in the treatment of PDB and GCT associated with PDB are shown in Table 21.

**Table 21:** Role of Denosumab in Paget's Disease

*Risk‐benefit balance*
From the evidence available, denosumab may be efficacious treating pain and reducing tumor size in GCT complicating PDB. There is little evidence supporting its use in PDB.
*Quality of evidence*
Very low
*Patient values and preferences*
Improvements in bone pain are highly valued by patients. Patients may be dissuaded by the need for repeated injections and risk of adverse events.
*Costs and use of resources*
Denosumab is considerably more expensive than bisphosphonates and involves repeated injections administered by a health care professional.
*Recommendation*
Denosumab may be considered for the treatment of GCT complicating PDB when the tumor is nonresectable. There is insufficient evidence to support the use of denosumab in the treatment of PDB, and it is not recommended for this indication.

### Predicting the response to treatment in Paget's disease

A large number of observational studies and clinical trials have been conducted in which biochemical markers of bone turnover have been measured before and after administration of various bisphosphonates in PDB. Indeed, most clinical trials of bisphosphonate therapy in PDB have used serum total ALP as the primary endpoint for efficacy.^66^ These studies have consistently shown that total ALP values and other biochemical markers of bone turnover are decreased by bisphosphonate therapy. It has been shown that the decrease is greater with nitrogen‐containing bisphosphonates as opposed to non‐nitrogen‐containing bisphosphonates, and that within the bisphosphonates, zoledronic acid is most effective at reducing total ALP.^66^

### Predicting the response of bone lesions

Al Nofal and colleagues conducted a systematic review and meta‐analysis of studies that compared changes in marker concentrations after bisphosphonate therapy with disease extent as assessed by quantitative radionuclide scintigraphy.^83^ Decreases in bone ALP concentrations after treatment have been observed after treatment with various bisphosphonates. However, in a meta‐analysis, bone ALP was a weak predictor of scintigraphic indices of disease extent after treatment (*r* = 0.24, 95% CI 0.004–0.457). Total ALP performed better than bone ALP but with confidence intervals that overlapped (*r* = 0.427, 95% CI 0.256–0.573), whereas PINP was the strongest predictor of the bone formation markers assessed (*r* = 0.704, 95% CI 0.559–0.808). The bone resorption markers uβCTX, sβCTX, uNTX, and sNTX also significantly predicted lesion extent assessed by scintigraphy after bisphosphonate treatment with values of 0.563, 95% CI 0.297–0.748 for uβCTX; 0.639, 95% CI for sβCTX 0.401–0.796; and 0.674, 95% CI 0.518–0.787 for uNTX.

The evidence summary and recommendations with regard to the use of biochemical markers in predicting the response of bone lesions to bisphosphonate treatment in PDB are shown in Table 22.

**Table 22:** Predicting Response of Bone Lesions to Bisphosphonate Treatment

*Risk‐benefit balance*
Biochemical markers of bone turnover can be easily assessed by analysis of blood or urine samples, and several markers of bone turnover are associated with scintigraphic extent of bone lesions after bisphosphonate therapy.
*Quality of evidence*
Very low
*Patient values and preferences*
Patients may value undergoing biochemical tests to predict the extent of PDB and response of bone lesions to bisphosphonates.
*Costs and use of resources*
The strongest predictor was PINP, but the confidence intervals overlapped with sβCTX, uNTX, and sNTX. These markers performed better than total ALP but are more expensive and not widely available.
*Recommendation*
Measurement of PINP is recommended to predict lesion extent, as defined by scintigraphy, after bisphosphonate therapy.

### Predicting the response of bone pain

Boudreau examined the relation between changes in bone pain in a series of 24 patients with PDB undergoing treatment with etidronate, mithramycin, or calcitonin in relation to bone scan appearances and changes in total ALP. They concluded that changes in blood flow as visualized on bone scan were the most reliable predictor of response of pain, although changes in total ALP and changes in bone scan static images after treatment also were associated with the response of pain.^125^ This study is of limited relevance to modern‐day treatment of PDB in view of the agents employed. A randomized placebo‐controlled trial of alendronic acid performed by Reid and colleagues^53^ demonstrated that the response of bone pain correlated poorly with reductions in serum total ALP and urinary NTX. At baseline, all patients had total ALP values at least twice the upper limit of normal, and 32 of 55 (58%) had pain thought to be due to PDB. After treatment, serum total ALP and uNTX values decreased by 78% and 86%, respectively, in the alendronic acid group but did not change significantly in the placebo group. Pain scores decreased by a mean (±SD) of –0.7 ± 0.5 in the placebo and –1.4 ± 0.3 in the alendronic acid group, a difference that was not significant (*p* = 0.4). A randomized trial by Siris and colleagues^112^ compared biochemical responses with responses of bone pain in PDB patients randomized to etidronate or risedronate sodium. All patients were required to have a total ALP value at least twice the upper limit of the reference range at baseline. At 6 months, ALP had decreased by 63.4% in the risedronate sodium group and 17% in the etidronate group (*p* < 0.001). The investigators reported that change in pain scores adjusted for analgesic use at 6 months showed no significant difference between groups (*p* = 0.07). In another randomized comparative trial of oral risedronate sodium 30 mg daily for 2 months and oral etidronate 400 mg daily for 6 months,^110^ risedronate sodium normalized total ALP in 73% of subjects at 6 months compared with 15% with etidronate (*p* < 0.001). In this study, pain scores (assessed by SF36) at 6 months reduced by about 3 points in the etidronate group (not significant) and 10 points in the risedronate sodium group (*p* < 0.01). The difference in pain scores between the groups (estimated by the 95% confidence intervals displayed on the graphs) was not significant.

The evidence summary and recommendations with regard to the use of biochemical markers in predicting the response of bone lesions to bisphosphonate treatment in PDB are shown in Table 23.

**Table 23:** Predicting Response of Bone Pain to Bisphosphonate Treatment

*Risk‐benefit balance*
Biochemical markers of bone turnover can be easily assessed by analysis of blood or urine samples, but these markers are poorly associated with response of bone pain to osteoclast inhibitors in PDB.
*Quality of evidence*
Very low
*Patient values and preferences*
Patients would value a test that could accurately predict the response of bone pain to bisphosphonate therapy.
*Costs and use of resources*
Total ALP is an inexpensive marker. Other specialized markers are considerably more expensive and not widely available.
*Recommendation*
Measurement of biochemical markers of bone turnover are not recommended a means of predicting the response of bone pain to osteoclast inhibitors in PDB.

### Predicting the response of other outcomes

There was no evidence upon which to identify predictors of change in quality of life, progression of deafness, fractures, bone deformity, or requirement for orthopedic surgery.

### Effects of nonpharmacological treatments in Paget's disease

No randomized trials were identified that investigated the effects of nonpharmacological treatments in PDB. The literature review identified several observational studies and case reports concerning the role of orthopedic surgery in PDB. Of these, we only considered series where the sample size was 10 or greater. No studies were identified that specifically investigated the role of physiotherapy, occupational therapy, or other nonpharmacological interventions in the management of Paget's disease.

### Surgical management of fractures

No randomized trials were identified with regard to the treatment of fractures in PDB, but the outcomes of surgical treatment have been reported in several observational studies, which for the most part, have been performed several decades ago.

Nicholas and colleagues evaluated clinical outcome of 23 PDB patients with fractures of the femur through affected bone referred to a specialist center for treatment.^126^ Various methods of treatment were used, including traction, intramedullary nails, and plating. Only 11 of 23 (47.8%) patients were felt to have a satisfactory outcome. Verinder evaluated clinical outcome of 89 fractures through affected bone in 67 patients with PDB who were treated over a 15‐year period in a single UK center.^127^ The femur was affected in 57 of 89 (64%) cases and the tibia in 22 of 89 (24.7%) and 10 of 89 (11.2%) in other sites. Various techniques were used, including joint replacement, internal fixation, traction, and long leg plaster. Most healed satisfactorily, but non‐union occurred in 8 of 11 (72.2%) patients with femoral neck fractures. Grundy^128^ evaluated the clinical outcome in 63 low‐trauma femoral fractures through affected bone in 48 patients presenting to a UK center over a 16‐year period. Various methods of management were used, including traction, plating, and intramedullary nails. Most fractures healed satisfactorily, but non‐union occurred in 11 of 11 (100%) femoral neck fractures. Bradley and Nade reviewed the outcome of 107 fractures of the femur through affected bone in 93 patients with Paget's disease over a 25‐year period from a center in New Zealand.^129^ The authors categorized subjects into those in whom the surgery was successful and those in whom it was not (which they termed failure). Failure was defined to be present if there was non‐union if the implant failed or if revision surgery was required. Femoral neck fractures had a high rate of failure (11 of 18 cases, 61.1%) as did subtrochanteric fractures (17 of 36; 47.2%), whereas failure was rare for fractures of the midshaft (1 of 24; 4.1%). Bidner and Finnegan^130^ reviewed the outcome of 35 femoral fractures occurring through affected bone over an 8‐year period in a Canadian center. Various methods of internal fixation were used. The authors commented that the results were generally satisfactory but that with subtrochanteric fractures, non‐union occurred in 3 of 10 (30%) of cases.

The evidence summary and recommendations with regard to surgical management of fractures in PDB are shown in Table 24.

**Table 24:** Surgical Management of Fractures in PDB

*Risk‐benefit balance*
The most commonly affected sites for fracture through Pagetic bone are the femur and tibia. Surgery may be technically difficult. Healing occurs normally in many patients, but the clinical outcome in proximal femoral fractures is poor. The benefit of fracture fixation in terms of pain relief and mobilization is likely to outweigh the risks of surgery.
*Quality of evidence*
Very low
*Patient values and preferences*
Patients highly value a positive clinical outcome after fracture fixation.
*Costs and use of resources*
The treatment costs for fracture fixation have not been evaluated but are likely to be similar to those in patients without PDB.
*Recommendation*
Surgery is recommended for fixation of fractures through affected bone in PDB, but the clinical outcome in femoral neck and subtrochanteric fractures is poor. There is insufficient information to recommend one type of surgical treatment over another.

### Total hip replacement surgery

We identified three case series of total hip replacements for osteoarthritis in patients with PDB. McDonald reviewed the outcome of cemented total hip replacements for osteoarthritis in 80 patients undergoing 91 hip replacements treated at a US referral center between 1969 and 1982.^131^ The femur was involved in 12 cases (13.2%), the acetabulum in 43 cases (47.3%), and both sites in 36 cases (39.6%). Heterotopic ossification was observed after surgery in 34 of 91 hips (37%), which the authors commented was much higher than expected in patients without PDB (4.7%). Radiographic evidence of prosthetic loosening was observed in 38 of 91 hips (41.7%). No association was observed between total ALP levels at the time of surgery or preoperative drug treatment with etidronate or calcitonin and the incidence of aseptic loosening. Revision was required in 14 of 91 hips (15.3%). The authors compared the likelihood of requiring revision for aseptic loosening in the PDB group with a series of 7222 patients without PDB undergoing hip replacement at the same center. There was no difference for up to 10 years, but subsequently requirement for revision was greater in the PDB subjects (approximately 40% compared with 5%, *p* < 0.001). The authors commented that the results of surgery were good or excellent in 74% of hips replaced. Wegryzn reviewed the clinical outcome of 39 cementless hip replacements in 32 patients undergoing surgery in a French center between 1992 and 2006.^(103)^ Heterotopic ossification occurred postoperatively in 22 of 39 hips (56%) and prosthetic loosening in 6 of 39 hips (15.3%). No patient had required revision surgery at the time of the review, which occurred on average 133.5 months (range 97 to 194 months) after surgery. Overall outcome (assessed by Harris hip score) was reported to be excellent in 27 patients (84%) and fair in 5 (18%).

Parvizi^132^ reviewed clinical outcome in 18 patients undergoing 19 uncemented total hip replacements in a US referral center between 1975 and 1996. In 18 of 19 (94%) cases, the serum total ALP was normal at the time of surgery. The outcome as assessed by Harris hip score was excellent in 16 cases (84.2%) and fair or good in 3 (15.8%). Heterotopic ossification occurred in 6 hips (31.5%) and aseptic loosening in 2 hips (10.5%). None of the patients had required revision surgery after an average follow‐up of 7.15 years (range 2 to 15).

### Total knee replacement surgery

Two case series of total knee replacement were identified. Gabel^(104)^ reviewed the outcome of total knee replacement (TKR) in 13 patients who had 16 joint replacements referred to a single US center between 1974 and 1986. Radiographic loosening was observed in two cases and one patient required revision surgery. The authors noted a functional improvement after surgery with a mean preoperative score of 33 points compared with 86 points postoperatively. They concluded that knee replacement was an effective procedure in patients with PDB. Lee reviewed the outcome of TKR in 21 knees from 20 patients with PDB undergoing treatment at a US center between 1978 and 1999.^105^ One patient required revision surgery for aseptic loosening after an interval of 10 years. The authors reported that all patients were satisfied with the procedures and felt that it had improved their quality of life.

The evidence summary and recommendations with regard to arthroplasty in the management of osteoarthritis in PDB are shown in Table 25.

**Table 25:** Total Knee and Hip Replacement for Osteoarthritis in PDB

*Risk‐benefit balance*
Total knee replacement (TKR) and hip replacement (THR) for osteoarthritis can be performed successfully in many patients with PDB with good results, although more data are available for THR. Heterotopic calcification occurs in a high proportion of patients undergoing THR and the risk of aseptic loosening may be slightly higher than in non‐Pagetic patients. The benefit of surgery is likely to outweigh the risks in most cases.
*Quality of evidence*
Very low
*Patient values and preferences*
Patients highly value the symptom relief and improvement in quality of life that a hip replacement may offer.
*Costs and use of resources*
The treatment costs for TKR and THR in PDB are likely to be similar to patients without PDB and this is recognized to be a cost‐effective option for patients with advanced osteoarthritis.
*Recommendation*
Total hip or knee replacements are recommended for patients with PDB who develop osteoarthritis in whom medical treatment is inadequate. There is insufficient evidence to recommend one type of surgical approach over another for either site.

### Osteotomy

Osteotomy is a recognized strategy for correction of bone deformity and improvement of pain in PDB. We failed to identify any studies in which osteotomy was compared with other treatment modalities and so the GDG was unable make recommendations on the role of this technique to be used as opposed to other surgical approaches.

Parvizi^107^ reviewed the outcome of 25 osteotomies in 22 patients with Paget's disease referred to a single US center. The indication for osteotomy was pain secondary to OA in 20 limbs, stress fractures in three, and deformity in two. The most common site was the tibia (*n* = 16) followed by the femur (*n* = 8) and radius (*n* = 1). Healing occurred in the vast majority of procedures (23 of 25), with an average time to union of 6 months, but this was significantly longer in metaphyseal (average 240 days, range 120 to 360) than diaphyseal osteotomies (average 150 days, range 60 to 360). Two patients had delayed union. Patient satisfaction was reported as excellent or good in 12 patients (60%), fair in 6 (30%), and poor in 2 (10%).

Roper and colleagues^133^ reviewed the results of osteotomy of the intertrochanteric region of femur in 14 patients treated at a single UK center. The indication for treatment was pain associated with OA of the hip joint in all cases. The authors reported that functional improvement had occurred in 12 of 13 (92.3%) patients and pain improved in 11 of 13 (84.6%), although details of the method of assessment of pain and function were not provided.

The evidence summary and recommendations with regard to osteotomy in the management of osteoarthritis in PDB are shown in Table 26.

**Table 26:** Osteotomy

*Risk‐benefit balance*
Osteotomy can be performed successfully with good results in many patients with PDB of the femur and tibia with good results. The benefit of surgery is likely to outweigh the risks in most cases.
*Quality of evidence*
Very low
*Patient values and preferences*
Patients highly value the symptom relief that osteotomy may provide in osteoarthritis.
*Costs and use of resources*
The treatment costs for osteotomy are likely to be lower than those of a total joint replacement.
*Recommendation*
Osteotomy may be considered for patients with PDB who develop osteoarthritis in whom medical treatment is inadequate, but there is insufficient evidence to make a recommendation on when this technique should be used as opposed to other surgical procedures such as arthroplasty.

### Spinal surgery

Jorge‐Mora and colleagues^106^ conducted a systematic review of patients undergoing surgical treatment of the spine in Paget's disease and identified 17 studies all of which described single case reports. The most common indication for surgery was spinal cord compression (*n* = 8), spinal stenosis (*n* = 6), and low back pain. The most common procedure was laminectomy (*n* = 12), although this was sometimes combined with other surgical procedures. Improvement (full or partial) was noted to occur in 14 of 17 cases.

The evidence summary and recommendations with regard to spine surgery in the management of PDB are shown in Table 27.

**Table 27:** Spinal Surgery in PDB

*Risk‐benefit balance*
Spine surgery can be performed successfully with good results in patients with PDB. The benefit of surgery is likely to outweigh the risks in most cases.
*Quality of evidence*
Very low
Patient values and preferences
Patients highly value the symptom relief and improvement in neurological symptoms that spine surgery may provide.
*Costs and use of resources*
The treatment costs for spine surgery are considerable, but in many cases the procedure may be cost‐effective.
*Recommendation*
Spine surgery may be considered for patients with PDB who develop spinal stenosis and spinal cord compression.

## Summary

This guideline is the result of a comprehensive systematic review on the diagnosis and management of PDB, which considered both pharmacological and nonpharmacological treatment options. A summary of the recommendations made are shown in Table 28.

**Table 28:** Summary of Recommendations

Investigation or indication	Recommendation	Conditional recommendation	Insufficient evidence
Diagnosis of PDB			
X‐rays	X‐rays of abdomen, skull, facial bone, and tibia recommended	–	–
Radionuclide bone scans	To fully determine extent of metabolically active disease	–	–
MRI and CT	Not recommended for diagnosis	May be considered to evaluate complications	–
ALP	First‐line biochemical test for metabolically active PDB in combination with LFT	–	–
PINP, BALP, NTX	–	Second‐line tests when suspicion of metabolically active disease is high and ALP is normal	–
Bisphosphonate treatment			
Bone pain	Recommended for the treatment of bone pain	–	–
Quality of life	–	–	Insufficient evidence; treatment not recommended
Fracture prevention	–	–	Insufficient evidence; treatment not recommended
Progression of osteoarthritis	–	–	Insufficient evidence; treatment not recommended
Progression of hearing loss	–	–	Insufficient evidence; treatment not recommended
Blood loss during elective orthopedic surgery	–	–	Insufficient evidence; treatment not recommended
Bone deformity	–	–	Insufficient evidence; treatment not recommended
Neurological symptoms	–	Calcitonin or bisphosphonates may be considered as part of the treatment package	–
Asymptomatic patients with increased metabolic activity	–	Bisphosphonates may be considered, but clinical benefit unclear	–
Neoplastic transformation	–	–	Insufficient evidence; treatment not recommended
Adverse effects of bisphosphonates	Patients can be reassured about the favorable adverse event profile	–	–
Treatment strategy			
Symptomatic or intensive bisphosphonate treatment	Treatment goal should be to control bone pain rather than normalize ALP	–	–
Route of neridronate administration	Intravenous and intramuscular both recommended	–	–
Other treatments			
Calcitonin for bone pain	–	May be considered for short‐term treatment of bone pain	–
Denosumab for treatment of PDB	–	–	Insufficient evidence; treatment not recommended
Denosumab for giant cell tumor	–	May be considered for treatment of giant cell tumor that is unresectable	–
Predicting response to treatment			
Predicting response of bone lesions	Measurement of PINP recommended to predict lesion extent defined by scintigraphy after treatment	–	–
Predicting response of pain	Measurement of biochemical markers is not recommended as a means of predicting response of bone pain	–	–
Nonpharmacological treatments			
Fracture fixation	Surgery is recommended for fixation of fractures through Pagetic bone	–	–
Hip or knee arthroplasty	Recommended for patients with PDB with OA where medical treatment is inadequate	–	–
Osteotomy	–	May be considered for patients with PDB with OA where medical treatment is inadequate	–

**Figure 2:**
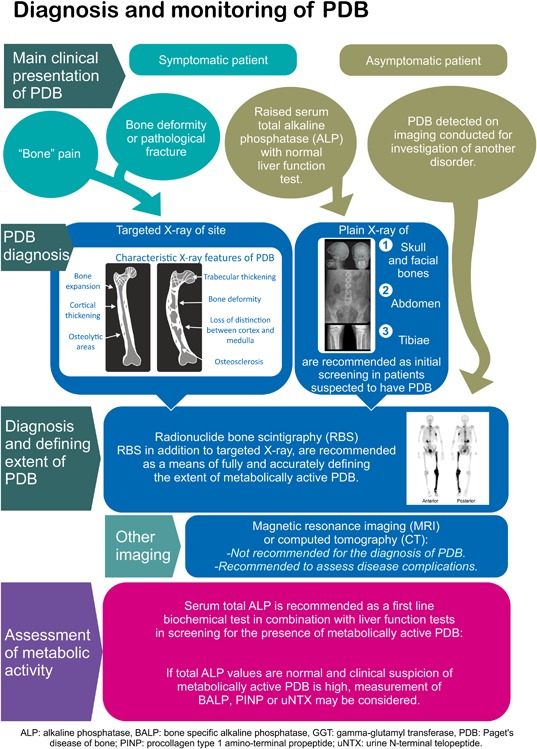
Diagnosis and monitoring of Paget's disease. ALP = total alkaline phosphatase; BALP = bone‐specific alkaline phosphatase; PINP = procollagen type I N‐terminal propeptide; uNTX = urinary cross‐linked N‐terminal telopeptide of type I collagen.

**Figure 3:**
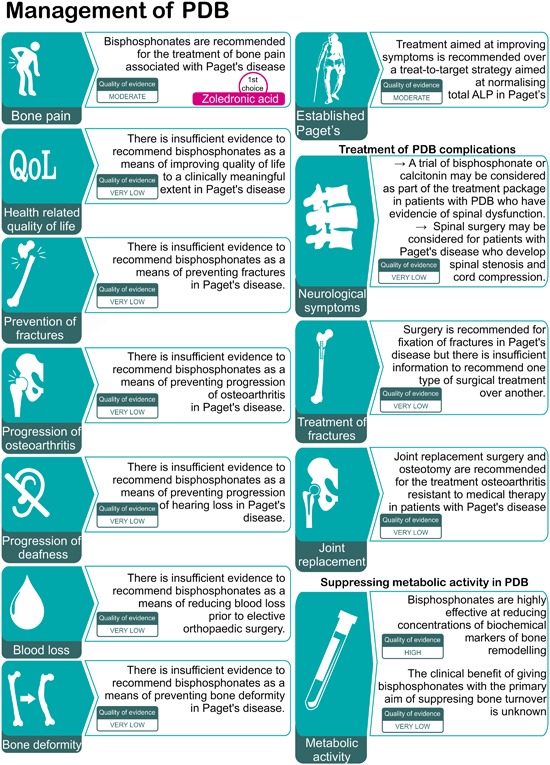
Management of Paget's disease.

A graphical summary of the recommendations for diagnosis and assessment of PDB is shown in Fig. 2 and for the management of PDB in Fig. 3.

Although we have made recommendations in 12 areas and conditional recommendations in five, the GDG noted that for several outcomes of clinical importance to patients, there was insufficient evidence to answer the questions posed in this guideline due to the fact that most clinical trials in PDB had been short term and focused on biochemical markers as the primary outcome, rather than patient‐reported outcome measures. Accordingly, the GDG felt that further research into PDB is warranted and identified the following topics as areas where research would be warranted (Table 29).

**Table 29:** Clinical Questions to Be Prioritized for Further Research in PDB

Risk and benefits of treating asymptomatic patients with PDB Effects of treatment on complications Role of genetic profiling in the diagnosis of PDB and prediction of complications Clinical outcome of joint replacement surgery and osteotomy in the modern era Clinical outcome after fracture fixation in the modern era Effects of nonpharmacological treatments other than surgery

## Disclosures

MCZ reports personal fees from Amgen, Eli Lilly, Shire, and Kyowa Kirin, outside the submitted work. NG reports personal fees from Amgen, UCB, Eli Lilly, and Alexion outside the submitted work. MKJ reports personal fees from Amgen outside the submitted work. KS, RGGR, SPT, and RMF report that they are trustees of the Paget's Association. DW reports that she is an employee of the Paget's Association. CC reports that he is president of the International Osteoporosis Foundation. RW reports that she is an employee of International Medical Press. SHR reports receiving research grants to his institution from Eli Lilly, Amgen, and UCB outside the submitted work and having received consultancy fees on behalf of his institution from Novartis outside the submitted work. The other authors state that they have no conflicts of interest.

## Supporting information

Supporting Data S1.
