# Supplementary material for: Diagnosis and Management of Paget's Disease of Bone in Adults: A Clinical Guideline
Source: J Bone Miner Res. 2019 Feb 25;34(4):e3657. doi: 10.1002/jbmr.3657 (PMC6522384; doi:10.1002/jbmr.3657)
Supplement: Supplementary file 1 — Supporting Data S1. [file JBMR-34-579-s001.docx]

**Supplementary Material**

**Diagnosis and Management of Paget’s disease of Bone in Adults:**

**A clinical guideline**

Stuart H Ralston, Luis Corral-Gudino, Cyrus Cooper Roger M Francis, William D Fraser, Luigi Gennari, Nuria Guanabens, M Kassim Javaid, Robert Layfield, Terence W O’Neill, R Graham G Russell, Michael D Stone, Keith Simpson, Diana Wilkinson, Ruth Wills, M. Carola Zillikens, and Stephen P Tuck.

**Appendix 1**

**Key questions used to develop the guideline.**

The guideline was developed using a series of key questions as summarised below.

| **1. Which measurements or tools are effective in the identification and diagnosis of Paget’s disease?** | |
| --- | --- |
| *Population* | Individuals being assessed for possible PDB |
| *Test* | Radiographs, radionuclide bone scan, MRI, CT scans, biochemical markers of bone metabolism, genetic markers, clinical examination. |
| *Comparisons* | Comparison between tests |
| *Outcomes* | Diagnostic accuracy (sensitivity, specificity, positive or negative predictive value). |
| *Section* | 4.1-4.4 |

| **2. Which diagnostic measurements or tools are effective in predicting the clinical response to treatment?** | |
| --- | --- |
| *Population* | Patients with Paget’s disease undergoing treatment |
| *Test* | Radiographs, radionuclide bone scan, MRI, CT scans, biochemical markers of bone metabolism, genetic markers, clinical evaluation |
| *Comparisons* | Treated patients who have no evaluation |
| *Outcomes* | Improvement in bone pain, quality of life, prevention of fractures, prevention of deformity, prevention of deafness biochemical markers of bone turnover, orthopaedic surgical procedures, tinnitus, audiometry, mortality, adverse events, radiographic change, histology, biochemical markers, sarcoma, spinal stenosis, neurological symptoms, cardiac failure, gout, blood lost at surgery, outcome of surgery, prosthetic loosening |
| *Section* | 8.1-8.4 |

| **3. Indications for drug treatment in Paget’s disease?** | |
| --- | --- |
| *Population* | Patients with PDB who are asymptomatic versus those who are symptomatic |
| *Interventions* | Bisphosphonates, denosumab, calcitonin, analgesics, NSAID, orthopaedic surgical procedures, non-pharmacological interventions, |
| *Comparisons* | No intervention, or placebo, comparison of interventions, or combinations of |
| *Outcomes* | Biochemical markers of bone turnover, bone pain, quality of life, fractures, orthopaedic surgical procedures, deafness, tinnitus, audiometry, mortality, adverse events, radiographic change, histology, biochemical markers, sarcoma, spinal stenosis, neurological symptoms, cardiac failure, gout, blood lost at surgery, outcome of surgery, prosthetic loosening |
| *Section* | 5.1-5.10 |

| **4. What are the effects of drug treatment in Paget’s disease?** | |
| --- | --- |
| *Population* | Patients being treated for Paget’s disease |
| *Interventions* | Bisphosphonates, denosumab, calcitonin, analgesics, NSAID, orthopaedic surgical procedures, non-pharmacological interventions, |
| *Comparisons* | No intervention, or placebo, comparison of interventions, or combinations of intervention. |
| *Outcomes* | Biochemical markers of bone turnover, bone pain, quality of life, fractures, orthopaedic surgical procedures, deafness, tinnitus, audiometry, mortality, adverse events, radiographic change, histology, biochemical markers, sarcoma, spinal stenosis, neurological symptoms, cardiac failure, gout, blood lost at surgery, outcome of surgery, prosthetic loosening |
| *Section* | 5.1-5.10 |

| **5. For individuals prescribed pharmacological interventions, what is the optimal duration or mode of treatment?** | |
| --- | --- |
| *Population* | Patients being treated for Paget’s disease |
| *Interventions* | Duration of treatment, mode of treatment (e.g. continuous treatment, single treatment. intermittent treatment), treatment when bone turnover rises, treatment on relapse or continuous |
| *Comparisons* | Any alternative mode or duration of intervention |
| *Outcomes* | Biochemical markers of bone turnover, bone pain, quality of life, fractures, orthopaedic surgical procedures, deafness, tinnitus, audiometry, mortality, adverse events, radiographic change, histology, biochemical markers, sarcoma, spinal stenosis, neurological symptoms, cardiac failure, gout, blood lost at surgery, outcome of surgery, prosthetic loosening. |
| *Section* | 6.1-6.2 |

| **6. What are the effects of non drug treatment in Paget’s disease?** | |
| --- | --- |
| *Population* | Patients being treated for Paget’s disease |
| *Interventions* | Surgery, physiotherapy, occupational therapy, diet any other non-pharmacological therapy, |
| *Comparisons* | No intervention, or placebo, comparison of interventions, or combinations of intervention. |
| *Outcomes* | Biochemical markers of bone turnover, bone pain, quality of life, fractures, orthopaedic surgical procedures, deafness, tinnitus, audiometry, mortality, adverse events, radiographic change, histology, biochemical markers, sarcoma, spinal stenosis, neurological symptoms, cardiac failure, gout, blood lost at surgery, outcome of surgery, prosthetic loosening |
| *Section* | 9.1-9.5 |

**Appendix 2**

**Search methods and study flow diagrams.**

Search method for identification of studies

We carried out electronic searches in the following databases:

1. Embase (Embase 1974 to 2016 Week 34)

2. Medline (Epub Ahead of Print, In-Process & Other Non-Indexed Citations, Ovid MEDLINE(R) Daily and Ovid MEDLINE(R) 1946 to Present)

The search results were reviewed to identify those relevant articles based on title and abstract. Full manuscripts of the selected articles were retrieved for further assessment based on full text. Review articles, case reports case series of less than 10 individuals and abstract summaries were excluded. For the included articles, the following data was extracted where relevant:

- Study identification (author, title, year of publication, journal)
- Characteristics of the study (study design, method of randomization, outcome)
- Characteristics of participants (number and gender, disease characteristics [monostotic vs polyostotic disease, symptomatic vs asymptomatic patients, severe vs mild disease, diagnosed vs undiagnosed], exclusion post-randomization and reasons for exclusion, participants assessed, withdrawals and reasons for withdrawals)
- Characteristic of intervention (measurement or tool analysed, comparator, co-interventions)
- Outcome data

## **Key question 1. Which measurements or tools are effective in the identification and diagnosis of Paget’s disease?**

| 1. exp bone disease/  2. 1 and paget$.mp.  3. (paget$ adj10 bone$).mp.  4. exp Paget bone disease/  5. osteitis deformans.mp.  6. ostitis deformans.mp.  7. or/2-6  8. diagnosis.ti,ab.  9. identification.ti,ab.  10. measurement.ti,ab.  11. assessment.ti,ab.  12. imaging.ti,ab.  13. radiograph$.ti,ab.  14. biochemical marker$.ti,ab.  15. genetic marker$.ti,ab.  16. scan$.ti,ab.  17. or/8-15  18. exp animals/ not humans.sh.  19. 17 not 18  20. case report.ti,ab.  21. 19 not 20  22. 7 and 21  23. limit 22 to english language  24. remove duplicates from 23 |  |
| --- | --- |

## **Key question 2. Which diagnostic measurements or tools are effective in predicting the clinical response to treatment?**

| 1. exp bone disease/  2. 1 and paget$.mp.  3. (paget$ adj10 bone$).mp.  4. exp Paget bone disease/  5. osteitis deformans.mp.  6. ostitis deformans.mp.  7. or/2-6  8. measurement.ti,ab.  9. assessment.ti,ab.  10. imaging.ti,ab.  11. radiograph$.ti,ab.  12. biochemical marker$.ti,ab.  13. genetic marker$.ti,ab.  14. scan$.ti,ab.  15. treatment.ti,ab.  16. response.ti,ab.  17. clinical response.ti,ab.  18. prediction.ti,ab.  19. prevention.ti,ab.  20. or/8-19  21. exp animals/ not humans.sh.  22. 20 not 21  23. case report.ti,ab.  24. 22 not 23  25. 7 and 24  26. limit 25 to english language  27. remove duplicates from 26 |  |
| --- | --- |

## **Key question 3. What are the indications for drug treatment in PDB?**

| 1. exp bone disease/  2. 1 and paget$.mp.  3. (paget$ adj10 bone$).mp.  4. exp Paget bone disease/  5. osteitis deformans.mp.  6. ostitis deformans.mp.  7. or/2-6  8. stratification.ti,ab.  9. identification.ti,ab.  10. asymptomatic.ti,ab.  11. symptomatic.ti,ab.  12. treatment.ti,ab.  13. intervention.ti,ab.  14. surgery.ti,ab.  15. orthopaedic.ti,ab.  16. pharmacological.ti,ab.  17. combination.ti,ab.  18. non-pharmacological.ti,ab.  19. or/8-18  20. exp animals/ not humans.sh.  21. 19 not 20  22. case report.ti,ab.  23. 21 not 22  24. 7 and 23  25. limit 24 to english language  26. remove duplicates from 25 |  |
| --- | --- |

## **Key question 4. What are the effects of drug treatment in Paget’s disease?**

| 1. exp bone disease/  2. 1 and paget$.mp.  3. (paget$ adj10 bone$).mp.  4. exp Paget bone disease/  5. osteitis deformans.mp.  6. ostitis deformans.mp.  7. or/2-6  8. treatment.ti,ab.  9. intervention,ti,ab.  10. randomized.ti,ab.  11. pharmacological.ti,ab.  12. combination.ti,ab.  13. effectiveness.ti,ab.  14. adverse effect$.ti,ab.  15. side effect$.ti,ab.  16. bone pain,ti.ab.  17. quality of life.ti,ab.  18. fracture adj5 prevention.ti,ab.  19. deformity.ti,ab.  20. hearing.ti,ab.  21. deafness.ti,ab.  22. or/8-21  23. exp animals/ not humans.sh.  24. 22 not 23  25. 7 and 24  26. limit 25 to english language  27. remove duplicates from 26 |  |
| --- | --- |

##

## **Key question 5. For individuals prescribed pharmacological interventions, what is the optimal duration or mode of treatment?**

| 1. exp bone disease/  2. 1 and paget$.mp.  3. (paget$ adj10 bone$).mp.  4. exp Paget bone disease/  5. osteitis deformans.mp.  6. ostitis deformans.mp.  7. or/2-6  8. treatment.ti,ab.  9. intervention.ti,ab.  10. duration.ti,ab.  11. posology.ti,ab.  12. mode of administration.ti,ab.  13. continuous.ti,ab.  14. intermittent.ti,ab.  15. relapse.ti,ab.  16. pharmacological.ti,ab.  17. combination.ti,ab.  18. effectiveness.ti,ab.  19. quality of life.ti,ab.  20. fracture adj5 prevention.ti,ab.  21. bone pain.ti,ab.  22. randomized.ti,ab.  23. observational.ti,ab.  24. or/8-23  25. exp animals/ not humans.sh.  26. 24 not 25  27. 7 and 26  28. limit 27 to english language  29. remove duplicates from 28 |  |
| --- | --- |

## **Key question 6. Which are the effects of non-drug treatment in Paget’s disease?**

| 1. exp bone disease/  2. 1 and paget$.mp.  3. (paget$ adj10 bone$).mp.  4. exp Paget bone disease/  5. osteitis deformans.mp.  6. ostitis deformans.mp.  7. or/2-6  8. treatment.ti,ab.  9. surgery.ti,ab.  10. orthopaedic.ti,ab.  11. non-pharmacological.ti,ab.  12. physiotherapy.ti,ab.  13. occupational therapy.ti,ab. (or occupational therapy.fs.)  14. diet.ti,ab.  15. lifestyle.ti,ab.  16. effective$.ti,ab.  17. quality of life.ti,ab.  18. randomized.ti,ab.  19. observational.ti,ab.  20. or/8-19  21. exp animals/ not humans.sh.  22. 20 not 21  23. 7 and 22  24. limit 23 to english language  25. remove duplicates from 24 |  |
| --- | --- |

**Appendix 3.**

**Stakeholders that were invited to comment on the guideline**

|  | **Response** | **Comments received** | **Outcome** |
| --- | --- | --- | --- |
| *American College of Rheumatology* | No | No |  |
| *American Orthopedic Association* | No | No |  |
| *American Society of Bone and Mineral Research* | Yes | No | Guideline endorsed |
| *Association of Clinical Biochemists (UK)* | No | No |  |
| *American Association of Clinical Chemistry (UK)* | No | No |  |
| *Australia & New Zealand Bone and Mineral Society* | Yes | No |  |
| *Bone Research Society, UK* | Yes | Yes | Guideline endorsed |
| *British Orthopaedic Association* | No | No |  |
| *European Calcified Tissues Society* | Yes | Yes | Guideline endorsed |
| *Endocrine Society* | No | No |  |
| *British Society of Rheumatology* | Yes | Yes |  |
| *British Geriatic Society* | Yes | Yes | Guideline endorsed |
| *Chinese Society of Osteoporosis and Bone Mineral Research* | No |  |  |
| *Indian Society For Bone & Mineral Research* | No |  |  |
| *International Chinese Musculoskeletal Research Society* | No |  |  |
| *International Osteoporosis Foundation* | Yes | No | Guideline endorsed |
| *Japanese Society for Bone and Mineral Research* | No |  |  |
| *Patient members of the Paget Association (UK)* | Yes | Yes | Guideline endorsed |
